# Assessment of large‐scale spatial variation in age‐specific survival and age at first breeding in a long‐lived species

**DOI:** 10.1111/1365-2656.70291

**Published:** 2026-06-05

**Authors:** Matia Haïm Muller, Fabian R. Ketwaroo, Wolfgang Fiedler, Olaf Geiter, Christof Herrmann, Michael Schaub

**Affiliations:** ^1^ Population Biology Research Unit Swiss Ornithological Institute Sempach Switzerland; ^2^ Centre for Animal Marking Max Planck Institute of Animal Behavior Radolfzell Germany; ^3^ Bird Ringing Centre Institute of Avian Research “Vogelwarte Helgoland” Wilhelmshaven Germany; ^4^ Hiddensee Bird Ringing Centre Agency for Environment, Nature Conservation, and Geology MV Güstrow Germany

**Keywords:** age at first breeding, age‐dependence, *Ciconia ciconia*, spatial variability, survival, synchrony

## Abstract

1. Age‐dependent survival and age at first breeding are key demographic parameters that drive population dynamics. Although crucial for understanding population trends and informing region‐specific conservation strategies, the large‐scale spatial variability of these demographic parameters, as well as how temporal fluctuations are correlated across space (i.e. spatial synchrony), are poorly known. This is particularly the case in long‐lived species characterised by complex, age‐dependent life cycles.
2. White storks (*Ciconia ciconia*) from Germany provide an opportunity to study spatial variation in age‐dependent survival and age at first breeding as they are long‐lived and extensively monitored through long‐term ringing programmes across the whole country. The contrasting population trends observed between western (increase) and eastern Germany (slight decrease), between which migratory flyways differ, suggest substantial spatial variation in demographic parameters.
3. We analysed ringing, resighting and recovery data of 92,251 individuals collected from 2000 to 2023 across Germany, using the federal states (Bundesländer) as spatial units. We fitted three spatial multistate capture–recapture–recovery models that differed in whether and how spatial autocorrelation among spatial units was incorporated.
4. The spatial patterns of the demographic parameters were similar across the three fitted models, but the model that used flyway information for the structure of spatial autocorrelation performed best. Survival in all age classes was higher and age at first breeding lower in western than in eastern Germany, consistent with the respective population trends. Spatial variability in survival was higher in juveniles than in other age classes, and synchrony across space was found in juvenile survival, but not in the survival of older age classes.
5. The developed models with alternative formulations of spatial autocorrelation were useful to assess the structure of spatial variation in survival and age at first breeding. Our results highlight substantial large‐scale spatio‐temporal variation in demographic parameters within a long‐lived species, helping to explain spatial differences in population dynamics across the study area.

## Graphical abstract

Using nationwide data from over 92,000 individuals, we reveal large‐scale spatio‐temporal variation in age‐dependent survival and age at first breeding in a long‐lived species. By comparing alternative spatial models, we show that demographic variation follows a biologically meaningful spatial structure, with implications for population dynamics.

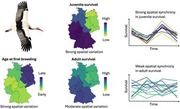

## 1 INTRODUCTION

Animal populations rise and fall according to the subtle balance of demographic processes (Allen & Hightower, 2010; Millon et al., 2019), with survival and age at first breeding being particularly influential in determining population trajectories (Caswell, 2001; Morris & Doak, 2002; Sæther & Bakke, 2000). As survival and age at first breeding are typically impacted by spatially heterogeneous environmental factors such as resource availability, predation pressure or population density, they vary across space (e.g. Péron et al., 2012; Sanz‐Aguilar et al., 2009). Ignoring spatial variation in the analyses of survival and age at first breeding can obscure important ecological patterns, as they arise as a consequence of local demographic processes (Gurevitch et al., 2016; Öst et al., 2016). The need for explicitly accounting for spatial variation in survival and age at first breeding, along with other demographic parameters, is increasingly recognised as essential for accurately characterising population dynamics (Chandler et al., 2018; Ozgul et al., 2006; Péron et al., 2012). Ultimately, it could help to implement site‐targeted conservation or management strategies (Morrison et al., 2022; Okamoto et al., 2020; Öst et al., 2016).

Knowledge of how survival and age at first breeding vary across large regions such as entire countries is currently limited. Some studies have mapped survival across large areas (e.g. Milleret et al., 2025; Saracco et al., 2010; Zhao, 2020), but they focused on a single age class and provided no information on age at first breeding. This is a key limitation when studying long‐lived species with age‐dependent survival and delayed and variable age at first breeding (Aubry et al., 2011). Two main knowledge gaps remain: (i) we lack knowledge about large‐scale spatial variation in age‐dependent survival and whether spatial survival patterns differ by age class—spatial variation may be greater in younger individuals, as seen with temporal variation (e.g. Souchay et al., 2013); and (ii) there has been no mapping of large‐scale spatial variation in age at first breeding. Moreover, studying spatial variation on a large scale while accounting for age also makes it possible to examine how temporal variation in survival is synchronised across space, how this synchrony decays with distance and how these features of synchrony (strength and decline with distance) vary with age, extending previous work on synchrony in survival that rarely accounted for age structure (e.g. Ghislain et al., 2024; Reiertsen et al., 2021; Schaub et al., 2015). This is important for understanding the scale and drivers of spatio‐temporal variation in survival within a species.

Advancing our knowledge about the spatial variation of demographic processes requires understanding how demographic values in one location relate to those in other locations. Tobler's first law of geography states that nearby locations tend to be more similar than distant ones, a pattern known as spatial autocorrelation (Tobler, 1970). This principle should apply directly to demographic parameters, because the environmental conditions that shape survival and age at first breeding are likely to be subject to spatial autocorrelation and therefore tend to generate similar demographic parameter values in neighbouring locations. Ignoring existent spatial autocorrelation has several consequences. First, ecological interpretations can be distorted, because populations in neighbouring locations are treated as independent units, attributing spatial differences to each location in isolation instead of recognising their shared environmental context. Second, assessments of uncertainty can be biased (Haining & Li, 2020). Third, the ability to estimate demographic parameters in sparsely sampled locations can be limited, because information cannot be borrowed across neighbouring locations, reducing the capacity to map demographic variation across large spatial scales. For all these reasons, spatial autocorrelation should be considered when analysing demographic variation across large spatial scales.

Estimating spatio‐temporal variation in age‐dependent survival and age at first breeding requires the availability of demographic data across large spatial and temporal scales. The white stork (*Ciconia ciconia*; hereafter stork) is a long‐lived bird that is known to exhibit strong age‐dependence in survival and in the probability to recruit into the breeding population (Barbraud et al., 1999; Doligez et al., 2004). This makes it particularly suitable for studying spatial variation in age at first breeding and in age‐specific survival, as well as for examining how these parameters vary through time and whether temporal fluctuations are synchronised across space. The German stork population is especially interesting: long‐standing contrasts in population trends between western and eastern Germany strongly suggest underlying spatial variation in key demographic parameters (Schimkat, 2022). Intensive monitoring of marked storks, including both live resightings and dead recoveries, has been conducted in Germany for decades, providing the life‐history data necessary to reliably study variation in demographic parameters across space and time.

In this study, we aim to characterise large‐scale spatio‐temporal variation in two key demographic parameters, age‐dependent survival and age at first breeding, using German storks from 2000 to 2023. To analyse the collected life‐history data, we used multistate capture–recapture–recovery (MCRR) models, a class of models widely used to estimate age‐ and time‐dependent survival and to infer age at first breeding (Lebreton et al., 2009), but that has not yet been extended to incorporate spatial autocorrelation. We first use a classical MCRR model that considers demographic parameters as independent between spatial units and then develop two novel MCRR models that incorporate spatial autocorrelation in age‐dependent survival and age at first breeding, differing in how they model spatial relationships between spatial units. Given observed population trends and existing knowledge on spatio‐temporal variation, we formulate four main predictions: (i) survival probabilities are higher in western Germany than in eastern Germany in all age classes; (ii) age at first breeding is lower in western Germany than in eastern Germany; (iii) spatial variation in survival declines with age; and (iv) there is synchrony in survival that declines with distance. By comparing three models with different spatial structures (including one without spatial autocorrelation), we assess how alternative ways of modelling spatial relationships between areas influence inference on age‐dependent survival and age at first breeding.

## 2 METHODS

### 2.1 Spatial resolution

**FIGURE 1:**
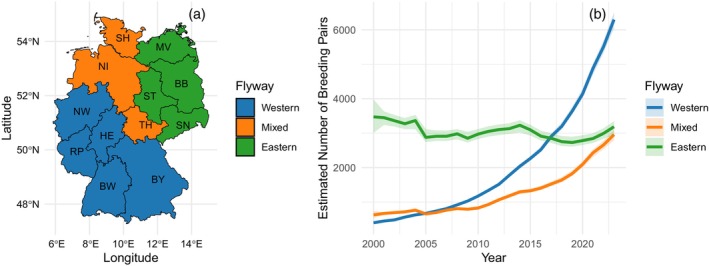
Division of Germany into 12 spatial units, with their corresponding abbreviations and assigned flyway categories (a). Development of the estimated number of white stork breeding pairs, with shaded bands showing 95% credible intervals (details in Appendix S1), in the different flyway areas (b).

The area in which storks were ringed covered the whole of Germany. We discretised space into 12 spatial units, primarily defined at the federal state (Bundesland) level. Four smaller federal states were merged with adjacent larger ones: Saarland with Rhineland‐Palatinate, Hamburg with Schleswig‐Holstein, Bremen with Lower Saxony and Berlin with Brandenburg (Figure 1a). Based on available information about the migration behaviour of storks from ringing and GPS tracking data (Cheng et al., 2019; Flack et al., 2016; Garthe, 2026; Rotics et al., 2016; Schmidt, 2010; Schüz, 1962; Shephard et al., 2013), each spatial unit was assigned to a category reflecting the dominant migratory flyway: western (area = 183,948 km^2^), eastern (92,796 km^2^) or mixed (80,890 km^2^), the latter indicating that storks use both flyways (Figure 1a). Storks using the western flyway travel via the Iberian Peninsula to reach sub‐Saharan West Africa, whereas those following the eastern flyway migrate through the Bosporus Strait toward sub‐Saharan East and Southern Africa (Shephard et al., 2013). In recent decades, a substantial proportion of western storks have begun overwintering in Europe and North Africa, particularly on the Iberian Peninsula, where they feed on landfills (Andrade et al., 2025; Cheng et al., 2019; Flack et al., 2016). Stork populations in the three flyway areas showed significantly different population trends between 2000 and 2023 (Figure 1b): they increased annually by 12.0% and 6.7% along the western and mixed flyway, respectively, and declined annually by 0.4% along the eastern flyway (see Appendix S1 for details on the estimation).

### 2.2 Life cycle of the white stork and resulting model assumptions

The stork displays the typical life‐history traits of a long‐lived species with high adult survival (Doligez et al., 2004), late age at first breeding (Barbraud et al., 1999) and low productivity (Tryjanowski & Sparks, 2008). After fledging, juveniles migrate to their wintering grounds. Over the next few summers, they either remain there or return to the breeding grounds, gradually recruiting into the breeding population (Van den Bossche et al., 2002). During their early life, individuals experience strong age‐dependent variation in survival: it is low in the first year of life (Rotics et al., 2016; Schaub et al., 2004), then increases but does not reach adult levels before at least 2 years of age (Doligez et al., 2004), which means that at least three age classes should be considered to model survival accurately. To account for more variation, we distinguished four age classes for annual survival: from fledging to the age of 1 year (juvenile survival), from 1 to 2 years of age (1y survival), from 2 to 3 years of age (2y survival) and after 3 years of age (≥3y survival).

First breeding typically occurs between the ages of 2 (third calendar year) and 5 (sixth calendar year), although a few individuals may start as early as 1 year (second calendar year); first breeding after the age of 5 is rare (Barbraud et al., 1999; Itonaga, 2009). Therefore, we assumed five recruitment age classes, and that all storks start breeding at age 5 at the latest. In line with the species' usual breeding behaviour (Barbraud et al., 1999), we assumed that once recruited, white storks would breed every year until death.

While dispersal from the site of birth to the breeding site can cover long distances, storks usually remain faithful to their breeding area once they have recruited (Chernetsov et al., 2006; Itonaga et al., 2010). To model emigration from a spatial unit we therefore considered two classes, natal and breeding, with natal emigration occurring once between the sites of birth and of first breeding, and breeding emigration occurring once any time after recruitment. Both types of emigration were therefore permanent (i.e. no return to a spatial unit once it has been left).

### 2.3 Data collection

Numerous volunteer ringers have marked stork nestlings with alphanumeric rings throughout Germany and resighted them later when they were breeding. Some of the marked storks that have died have been found (regardless of the cause of death, and both in and outside Germany) and the ring reported to the national ringing offices. Data from 2000 to 2023 were compiled by the three German ringing schemes Helgoland, Hiddensee and Radolfzell. We derived individual capture histories containing information on the year in which each bird was ringed, resighted as a breeder (defined as observed during nest building, incubation or nestling feeding) and/or found dead (recovered). Each capture history was linked to the spatial unit where the individual was marked as a nestling. Resightings without specified breeding status were excluded, since lack of breeder records does not confirm non‐breeding.

We included birds that were ringed before year 2000 and were resighted as breeders in or after 2000. These birds were considered ‘ringed’ when first resighted in or after 2000, and their age at their first resighting was calculated and included in the analysis. All resightings of an individual as a breeder in different spatial units from the one in which it was ringed were omitted, because modelling movement explicitly between each spatial unit was not an objective of the study (rather we estimated emigration from spatial units without considering where emigrants ended up). All dead recoveries were retained, regardless of their location, including those reported outside Germany. Details about the number of ringed individuals, resightings and dead recoveries per spatial unit are provided in Table 1. The average ringing date, used as the annual reference point for survival estimates, was 17th June.

**TABLE 1:** List of spatial units, their codes, their areas, the flyway to which they belong and the number of ringed storks (2000–2022), resightings (2001–2023) and dead recoveries (2000–2023).

Spatial unit	Code	Area (km^2^)	Flyway	Ringed birds	Resightings	Dead recoveries
Baden‐Württemberg	BW	35,748	West	23,836	10,731	1730
Rhineland‐Palatinate & Saarland	RP	22,430	West	8012	2560	558
Bavaria	BY	70,542	West	5071	1628	415
Hesse	HE	21,116	West	5301	2123	419
North Rhine‐Westphalia	NW	34,112	West	3317	463	222
Thuringia	TH	16,202	Mixed	1034	61	53
Lower Saxony & Bremen	NI	48,129	Mixed	9514	1595	479
Schleswig‐Holstein & Hamburg	SH	16,559	Mixed	4102	858	262
Saxony	SN	18,450	East	4788	589	223
Saxony‐Anhalt	ST	20,465	East	9117	1162	398
Mecklenburg‐Western Pomerania	MV	23,295	East	7709	753	407
Brandenburg & Berlin	BB	30,546	East	10,450	1292	311
Total		357,592		92,251	23,815	5477

### 2.4 Spatial MCRR models

We transformed our data into MCRR matrices, with one matrix per spatial unit. We then defined a total of 16 states: (1) juvenile, alive; (2) 1y breeder, alive; (3) 2y breeder, alive; (4) ≥3y breeder, alive; (5) 1y recently dead; (6) 2y recently dead; (7) 3y recently dead; (8) ≥4y recently dead; (9) 1y non‐breeder, alive; (10) 2y non‐breeder, alive; (11) 3y non‐breeder, alive; (12) 4y non‐breeder, alive; (13) 1y emigrated (permanently moved out of the spatial unit), alive; (14) 2y emigrated, alive; (15) ≥3y emigrated, alive; (16) long dead (absorbing state for dead individuals). The first eight states correspond to observed states in the MCRR matrices, while the remaining eight represent unobservable states needed in the model for parameter estimation.

**FIGURE 2:**
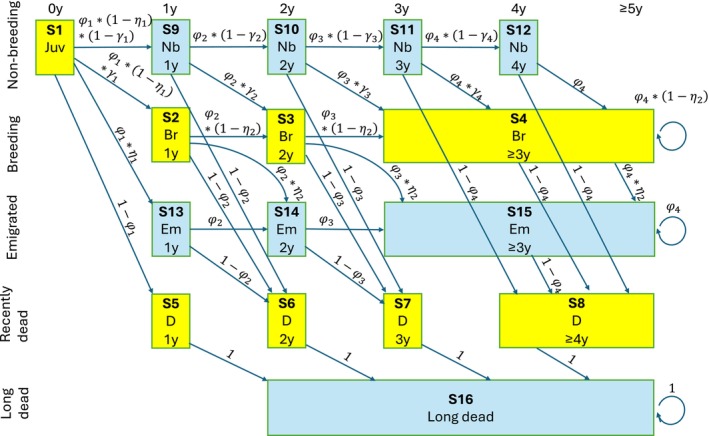
Graphical presentation of the state‐transition matrix of the three multistate capture–recapture‐recovery models. Shown are all possible transitions between the 16 states (S1–S16), categorised by age and status. Juv = Juvenile; 1y = 1‐year‐old; 2y = 2‐year‐old; 3y = 3‐year‐old; 4y = 4‐year‐old; Nb = Non‐breeding; Br = Breeding; Em = Emigrated; D = Recently dead. *φ* represents survival, *γ* recruitment and *η* emigration. Subscript numbers refer to the age class for survival and recruitment, and to the type of emigration: natal (1) or breeding (2). Colours indicate whether a state is observable (yellow) or unobservable (blue). Survival and recruitment were allowed to vary temporally and spatially, while emigration varied only spatially.

Two main components govern multistate models: the state‐transition matrix and the observation probabilities. The state‐transition matrix describes the transition probabilities of individuals from a given state in a given year to states in the next year. The transition probabilities are functions of the underlying demographic parameters to be estimated, that is age‐specific survival (φ), age‐specific probability to recruit into the breeding pool (γ) and permanent emigration (η) (Figure 2). Observation probabilities specify the probability of observing a marked individual in a given state during a given year. In our case, observation probabilities were the resighting probabilities (p) for breeders (states 2, 3 and 4) and the dead‐recovery probabilities (r) for recently dead individuals (states 5–8). The observation probabilities for the other states were 0.

We summarised our MCRR matrices into *m*‐arrays (Burnham, 1987), which aggregate data such that more computationally efficient model fitting is possible (Schaub & Badia‐Boher, 2025). Models using *m*‐arrays do not track individual identity but instead estimate, for each release cohort (i.e. the state–year combination in which an individual is encountered), the probabilities of being re‐encountered at any time in any state and of never being re‐encountered, using the following multinomial likelihood (Kéry & Schaub, 2012):

(1) $$m_{i,z}∼\text{Multinomial}\;\left(\pi_{i,z},R_{i,z}\right),$$

where $m_{i,z}=\left(m_{i,z,1},\ldots ,m_{i,z,T+1}\right)$ is the vector of the number of observations in each year and state of release cohort z (z=1,…,Z, with Z being the product of the numbers of release years and states) from the spatial unit i (i=1,…,12) and $R_{i,z}$ is the total number of individuals released in cohort z and unit i. The vector $\pi_{i,z}=$ $\left(\pi_{i,z,1},\ldots ,\pi_{i,z,Z+1}\right)$ contains the probabilities (one for each of the Z+1 possible outcomes) that an individual from unit i in cohort z is re‐encountered in a given state–year combination or is never re‐encountered. The elements in π are functions of the state‐transition parameters (Figure 2) and the observation probabilities.

We adapted our models to increase computational efficiency by removing all elements of π that were equal to 0 due to impossible transitions or referring to unobservable states from the multinomial likelihood (see Appendix S1 for more information).

We fitted three models (M1, M2, M3) that differed in the spatial autocorrelation structure they used for survival and recruitment, but not in all the other model parameters (emigration, resighting and recovery probabilities). In all these models, survival ($\varphi_{i,a_{\varphi },t}$) and recruitment ($\gamma_{i,a_{\gamma },t}$) were modelled as a function of spatial unit i, age class ($a_{\varphi }=1,\ldots ,4,a_{\gamma }=1,\ldots 5$) and time (t=1,…,23). To ensure that all storks start breeding at the latest when 5 years old, recruitment for age class $a_{\gamma }=5$ was fixed to one for all spatial units and times in all three models.

Model 1 (M1) assumed no spatial autocorrelation in survival and recruitment and estimated independent fixed spatial effects for each unit:

(2) $$\text{logit}\left(\varphi_{i,a_{\varphi },t}\right)=\mu_{i,a_{\varphi }}^{\varphi }+\zeta_{i,a_{\varphi },t}^{\varphi }$$

(3) $$\text{logit}\left(\gamma_{i,a_{\gamma },t}\right)=\mu_{i,a_{\gamma }}^{\gamma }+\zeta_{i,a_{\gamma },t}^{\gamma },$$

where $\mu_{i,a_{\varphi }}^{\varphi }$ and $\mu_{i,a_{\gamma }}^{\gamma }$ are respectively the logit transformed mean survival and recruitment per spatial unit i and age class a, $\zeta_{i,a_{\varphi },t}^{\varphi }$ and $\zeta_{i,a_{\gamma },t}^{\gamma }$ are the spatio‐temporal random effects that were modelled as $\zeta_{i,a_{\varphi },t}^{\varphi }∼\text{Normal}\;\left(0,\sigma_{i,a_{\varphi }}^{\varphi }\right)$ and $\zeta_{i,a_{\gamma },t}^{\gamma }∼\text{Normal}\left(0,\sigma_{i,a_{\gamma }}^{\gamma }\right)$, respectively, where $\sigma_{i,a_{\varphi }}^{\varphi }$ and $\sigma_{i,a_{\gamma }}^{\gamma }$ are the standard deviations.

Model 2 (M2) included spatial autocorrelation in survival and recruitment using a standard intrinsic conditional autoregressive (ICAR) model (Besag, 1974; see Appendix S1):

(4) $$\text{logit}\left(\varphi_{i,a_{\varphi },t}\right)=\mu_{a_{\varphi }}^{\varphi }+\epsilon_{i,a_{\varphi }}^{\varphi }+\zeta_{i,a_{\varphi },t}^{\varphi }$$

(5) $$\text{logit}\left(\gamma_{i,a_{\gamma },t}\right)=\mu_{a_{\gamma }}^{\gamma }+\epsilon_{i,a_{\gamma }}^{\gamma }+\zeta_{i,a_{\gamma },t}^{\gamma },$$

where $\mu_{a_{\varphi }}^{\varphi }$ and $\mu_{a_{\gamma }}^{\gamma }$ are respectively the logit transformed overall mean survival and recruitment per age class a, $\epsilon_{i,a_{\varphi }}^{\varphi }$ and $\epsilon_{i,a_{\gamma }}^{\gamma }$ represent spatial random effects that are modelled using an ICAR model, which ensures that they are spatially autocorrelated, and $\zeta_{i,a_{\varphi },t}^{\varphi }$ and $\zeta_{i,a_{\gamma },t}^{\gamma }$ are temporal random effects that are modelled as in M1. In an ICAR model, the definition of neighbour relationships among spatial units determines the structure over which spatial autocorrelation is imposed. For M2, we defined spatial units that share a common border as neighbours, thereby imposing spatial autocorrelation across all spatial units via the adjacency structure.

Model 3 (M3) also used an ICAR formulation for survival and recruitment, but structured spatial autocorrelation by flyway rather than across all units. $\varphi_{i,a_{\varphi },t}$ and $\gamma_{i,a_{\gamma },t}$ are modelled as in M2 (Equations 4 and 5). However, in contrast to M2, the neighbours are defined as spatial units sharing a common border *and belonging to the same flyway* (as defined in Section 2.1). As a result, spatial autocorrelation is restricted to within flyways rather than across the entire study area.

All other components (emigration, observation probabilities) were the same across all three models. Emigration probability $\eta_{i,a_{\eta }}$ was modelled for each class ($a_{\eta }$ = 1, 2) with an overall mean $\mu_{a_{\eta }}^{\left(\eta \right)}$ across all spatial units:

(6) $$\text{logit}\left(\eta_{i,a_{\eta }}\right)=\mu_{a_{\eta }}^{\eta }+\zeta_{i,a_{\eta }}^{\eta },$$

where $\zeta_{i,a_{\eta }}^{\eta }∼\text{Normal}\;\left(0,\sigma_{a_{\eta }}^{\eta }\right)$ accounts for variation between spatial units, with $\sigma_{a_{\eta }}^{\eta }$ as the standard deviation of the spatial variability for age class $a_{\eta }$. We did not include spatial autocorrelation in emigration because the spatial units differ strongly in size and shape, which strongly affects estimates of emigration probabilities independently of dispersal behaviour.

Resighting probability was modelled as a function of spatial unit, year and of whether an individual had been observed in the previous year, to account for potential immediate trap effects (Cachelou et al., 2026; Pradel, 1993). Since most dead recoveries are made by the public, we assumed that the dead‐recovery probability varies spatially due to differences in human population density, but not temporally. Therefore, we modelled dead‐recovery probabilities as constant over time but varying spatially, with spatial autocorrelation accounted for via an ICAR model (structure as in M2) and differing between age classes. A detailed description of the modelling of observation probabilities is provided in Appendix S1.

Within the model, we computed synchrony and age at first breeding as derived parameters. To quantify synchrony in survival fluctuations across space, we calculated for each age class independently the pairwise Pearson correlation coefficients of the time series $\zeta_{i,a_{\varphi },1:T}^{\varphi }$ between all spatial units (T being the number of years with survival estimates).

To obtain the mean age at first breeding in each unit over the study period, we first calculated the probability $\kappa_{i,a_{\gamma }}$ that a new‐born will start breeding in spatial unit i at each age class $a_{\gamma }$ =1,…,5, conditional on its survival until that age class (Pradel & Lebreton, 1999):
(7)

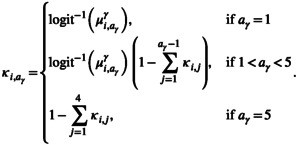




The mean age at first breeding per unit was then computed as the following weighted sum:

(8) $$A_{i}=\sum_{j=1}^{5}j\kappa_{i,j}.$$

Details about computation of overall (across all spatial units) mean age at first breeding and time‐dependent age at first breeding are given in Appendix S1.

We performed inference for the three models in the Bayesian framework using MCMC methods in NIMBLE (version 1.3; de Valpine et al., 2017). We used vague priors (Appendix S1) and ran each model (M1, M2, M3) for 600,000 iterations, with 4 chains, a burn‐in of 10,000 and a thinning rate of 1 in 600. All parameters of interest converged (R‐hat <1.1; Brooks & Gelman, 1998) in all three models. We performed model selection using the Widely Applicable Information Criterion (WAIC; Watanabe, 2010).

### 2.5 Ethics statement

This study was based on existing ringing, resighting and recovery data and did not involve any new animal handling or fieldwork by the authors. The original ringing activities were conducted by trained and licensed ringers under the coordination of the responsible German ringing centres (Hiddensee, Helgoland and Radolfzell) and under permits issued by the competent nature conservation authorities of the German federal states. No additional ethical approval was required for the analyses presented here.

## 3 RESULTS

### 3.1 Model comparison

**FIGURE 3:**
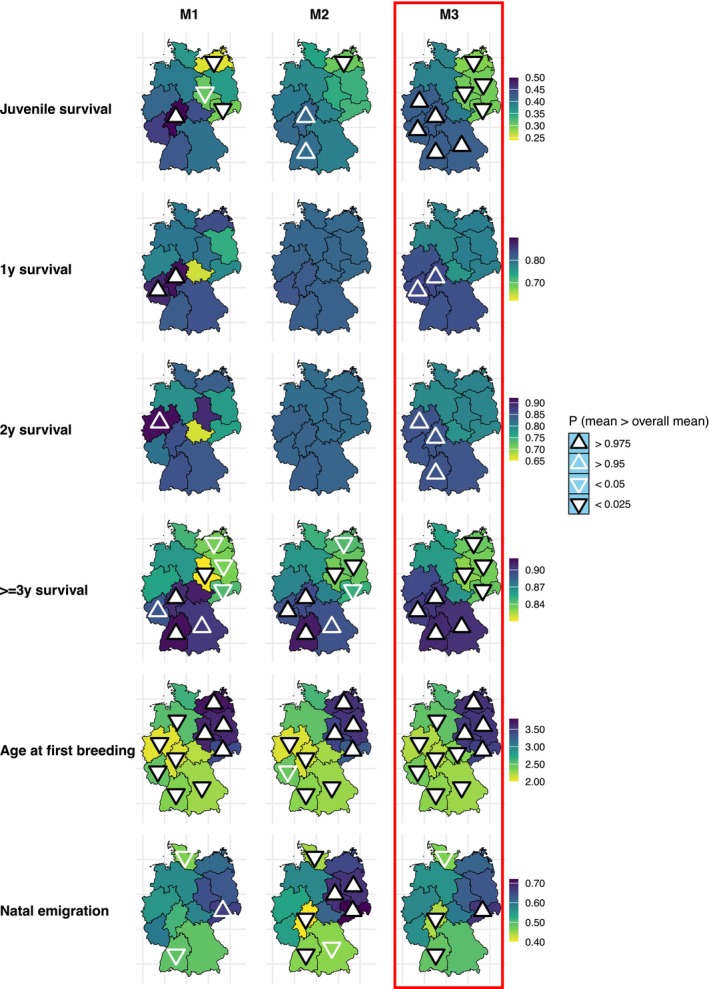
Maps of Germany showing posterior means of mean survival (four age classes), mean age at first breeding and emigration due to natal dispersal (natal emigration), for all three models: M1 (independent), M2 (standard ICAR) and M3 (flyway‐structured ICAR). Triangular symbols indicate spatial units with a high probability that their mean differs from the overall mean. Panels from the best model according to WAIC (M3) have a red box.

The spatial patterns of mean age‐specific survival and age at first breeding were similar across the three fitted models (Figure 3). Generally, survival tended to be higher in the west and lower in the east, and age at first breeding was lower in the west than in the east. Differences between models primarily concerned survival of juvenile, 1y and 2y individuals. M1 produced the most heterogeneous pattern, M2 produced the most homogeneous pattern and M3 produced a pattern that was more clearly structured by flyways (Figure 3). In general, parameter uncertainty was higher in M1 (without spatial autocorrelation) than in the two models accounting for spatial autocorrelation (Figure S1). Within these models, uncertainty was greater in M2 than in M3 for juvenile and adult survival and age at first breeding. However, the reverse was true for 1y and 2y survival (Figure S1).

Model selection indicated that M3 was better supported than M2, and that both M2 and M3 outperformed M1 (Table 2). We therefore present results from M3 in the following sections. Results from M1 and M2 are available in Appendix S3.

**TABLE 2:** Model selection results for the three multistate capture–recapture–recovery models fitted.

Model	ΔWAIC	lppd	pWAIC
M1 (independent)	25.67	−8598	907
M2 (standard ICAR)	8.36	−8585	912
M3 (flyway‐structured ICAR)	0.00	−8601	891

*Note*: Shown are the ΔWAIC (difference in WAIC between the most parsimonious and the current model), lppd (log posterior predictive density) and pWAIC (effective number of parameters).

### 3.2 Survival

The overall mean of annual survival probability across all spatial units and years was 0.369 (95% CRI 0.346–0.393) for juveniles, 0.802 (95% CRI 0.769–0.832) for 1y individuals, 0.820 (95% CRI 0.791–0.848) for 2y individuals and 0.873 (95% CRI 0.861–0.884) for ≥3y individuals (hereafter adults). Mean survival probabilities were higher in spatial units belonging to the western flyway (western Germany) than in units belonging to the eastern flyway (eastern Germany) across all age classes (Figure 3; Table S1). The units belonging to the mixed flyway had intermediate values for juveniles and adults, and the lowest mean values for 1y and 2y individuals (Figure 3; Table S1). The posterior probability of unit‐specific survival exceeding the overall mean was very high in western Germany and very low in eastern Germany, for both juveniles and adults (Figure 3; see Appendix S1 for the calculation of posterior probabilities).

Spatial variability in survival was higher in juveniles than in the older age classes, particularly in adults: the posterior probability that the range in survival across spatial units (maximum minus minimum survival estimate) was greater for juveniles than for 1y, 2y and adults was 0.73, 0.84 and 0.98, respectively. The posterior probability that the range in survival was greater for 1y than for 2y was 0.60, 0.71 for 1y than for adults and 0.64 for 2y than for adults.

**FIGURE 4:**
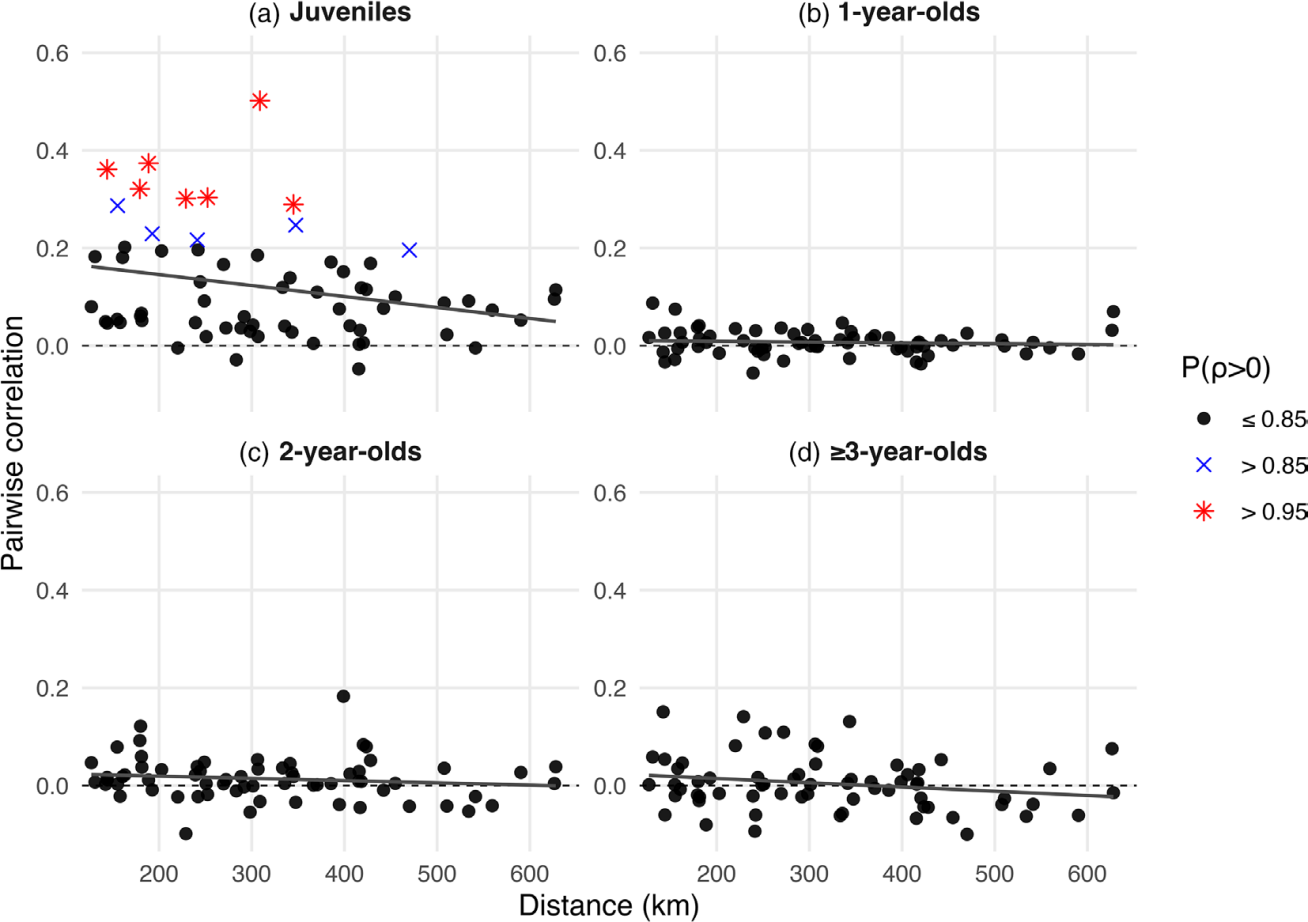
Illustration of how synchrony in survival declines with distance. Dots show posterior means of Pearson correlation coefficients of temporal fluctuations in survival between pairs of spatial units. These are plotted against distance between the spatial units. (a) Juveniles, (b) 1‐year‐old individuals, (c) 2‐year‐old individuals and (d) ≥3‐year‐old individuals. Estimates are from M3. The shape and colour of symbols indicate the probability of a positive pairwise correlation (see legend).

Juvenile survival in units belonging to the western flyway tended to increase over the study period, while there was no clear trend for units belonging to the eastern or mixed flyways (Figure S2). The annual fluctuations in survival were often larger in juveniles than in older age classes: the posterior probability that juveniles showed a larger temporal range than adults was ≥0.95 in six spatial units (Table S2). Temporal fluctuations in juvenile survival were synchronised between spatial units, with a tendency for decreasing synchrony with increasing distance (Figure 4a). No spatial synchrony was found in older age classes (Figure 4b–d).

### 3.3 Age at first breeding

The overall mean of age at first breeding across all spatial units was 2.69 (95% CRI 2.59–2.77) years. Unit‐specific mean ages at first breeding were higher in units belonging to the eastern than to the western or mixed flyway (Figure 3; Table S1). In mixed flyway units, ages at first breeding were closer to those from the western than to the eastern flyway (Figure 3; Table S1). The probability of exceeding the global mean was very high for units belonging to the eastern flyway and very low for units belonging to the western flyway (Figure 3).

Although age at first breeding exhibited annual fluctuations in some units (Figure S3; Table S1), no clear temporal trends emerged (Figure S3).

### 3.4 Emigration

The overall mean of natal emigration probability across all spatial units was 0.561 (95% CRI 0.491–0.630). Natal emigration was higher in eastern flyway units than in western ones (Figure 3; Table S1). The breeding emigration probability had an overall mean of 0.018 (95% CRI 0.006–0.030) and was consistently very low in all spatial units (Table S1).

## 4 DISCUSSION

The use of spatial MCRR models revealed substantial spatial variation in survival and age at first breeding in storks throughout Germany. In line with our first and second predictions, survival probabilities were higher in all age classes and first breeding occurred at younger ages in western Germany than further east. Consistent with our third prediction, spatial variation in survival was larger in juveniles compared to older age classes. Furthermore, consistent with our fourth prediction, there was a positive correlation between temporal fluctuations in survival that tended to weaken with distance, but only in juveniles. These findings emphasise the key role of spatial structure in shaping demographic processes.

Our work represents a significant advance in understanding how demographic parameters vary across age, space and time. We could generate (i) maps of age‐specific survival, which revealed that the strength of spatial variation differed between age classes, (ii) maps of age at first breeding, showing strong spatial differences in this key life‐history trait and (iii) estimates of synchrony in survival across a large and contiguous geographic area. We were thus able to address previously poorly investigated ecological questions concerning how demographic parameters vary with age, space and time. Our modelling approach provides a flexible and comprehensive framework for investigating spatio‐temporal variation in demography, which is particularly relevant for long‐lived species that exhibit strong age‐dependent demographic patterns.

An assumption of our model is that the dead‐recovery probability is the same for individuals who have emigrated and those who have not (Burnham, 1993). This assumption is likely to be adequately met in our study, as the spatial units are large and emigrants are therefore expected to end up mainly in nearby areas where the recovery probability is similar. The age‐specific survival estimates obtained in our study fall within the range reported by earlier non‐spatial studies on white storks (Cheng et al., 2019; Doligez et al., 2004; Rotics et al., 2016), supporting the plausibility of our results.

We compared three alternative models that differed in the spatial structure adopted for age‐specific survival and recruitment: an independent model (M1), a standard ICAR model (M2) and a flyway‐structured ICAR model (M3). Across all three models, spatial variation consistently pointed to higher survival and earlier age at first breeding in western Germany, indicating that this pattern did not depend on the specific spatial structure assumed. However, the independent model (M1) produced high uncertainty and heterogeneity and received the least support from the data (WAIC), showing that accounting for spatial autocorrelation is essential. In contrast, the standard ICAR model (M2) substantially reduced uncertainty but tended to over‐smooth parameter estimates, resulting in unrealistically homogeneous spatial patterns in survival for data‐sparse age classes such as 1y and 2y individuals. Over‐smoothing is a known feature of ICAR models (Duncan & Mengersen, 2020), and can be reduced by adopting spatial structure a priori. This was done in the flyway‐structured ICAR model (M3), where spatial smoothing was restricted to the defined flyways. The flyway‐structured ICAR performed best overall: it achieved the lowest WAIC among the three models, improved the representation of spatial variation in data‐sparse age classes and retained much higher precision than the independent model. It therefore provided a good balance between low uncertainty and spatial interpretability, suggesting that using a spatial structure grounded in the species' ecology can help obtain realistic spatial inference on demographic parameters.

We defined our spatial units according to administrative boundaries, encompassing relatively large areas. Spatial variation occurring at smaller spatial scales might have gone undetected given our spatial resolution. Our design is therefore more suited for examining spatial variation in regional averages rather than local extremes. Spatial analyses often use regular grids as spatial units to ease inference (e.g. Saracco et al., 2010; Socolar et al., 2025), while our study used administrative spatial units instead, which are irregularly shaped and vary in size. This spatial stratification was used because it corresponds to the spatial scale at which the data were collected. Since management actions in Germany are organised at the level of political‐administrative entities such as the Bundesländer, getting information for these actions at the same spatial scale is an advantage.

Our findings allow interesting insights into the biological mechanisms underlying spatial demographic variation. The population growth rate of long‐lived species is particularly sensitive to changes in survival of reproducing individuals and age at first breeding (Sæther & Bakke, 2000; Schaub et al., 2004). It is therefore likely that the higher survival probabilities and the earlier start of breeding of storks in western Germany are the main factors contributing to the difference in population growth between western and eastern populations. A rigorous test of this hypothesis would require estimates of productivity and the use of retrospective population analyses (Koons et al., 2016).

We believe that differences in survival and age at first breeding between storks of the western and eastern flyway can be explained by the different migration behaviour. Migration distance has often been shown to have a negative impact on survival among various bird species (e.g. Lok et al., 2011), including storks (Cheng et al., 2019; Rotics et al., 2017). This finding aligns with the observed lower survival of storks in eastern Germany, who migrate much farther than those from western Germany. As juvenile birds are generally more sensitive to environmental (e.g. Oro et al., 2010) and migratory challenges (e.g. Rotics et al., 2016) than adults, their survival may vary more among migratory strategies, which could explain the greater spatial variation we observed in stork juvenile survival. Migration distance has also been shown to affect age at first breeding, with longer migration distances being associated with later age at first breeding in long‐lived bird species (Cayuela et al., 2025), a pattern that is consistent with the later age at first breeding we observed in eastern Germany in our storks. Indeed, long migration journeys can reduce body condition, which is known to delay recruitment (Weimerskirch, 1992). Furthermore, individuals that winter closer to breeding sites tend to arrive earlier than those migrating longer distances (as observed in our storks; Schimkat, 2022), and earlier seasonal arrival in breeding grounds has been linked to earlier recruitment in long‐lived species (Becker et al., 2008). Further research in long‐lived migratory species should investigate the direct role of migration strategies in generating spatial differences in survival and in age at first breeding, for example by studying storks from areas where the western and eastern flyways overlap. Alternatively, the spatial differences in survival and age at first breeding could have arisen from density‐dependent processes, given that population densities were generally higher in eastern than in western Germany during most of the study period and that density is known to decrease survival and delay age at first breeding in different species (Bonenfant et al., 2009; Katzenberger et al., 2021). However, our results provide opposite evidence: in western Germany, survival did not decline (juvenile survival even tended to increase; see Figure S2) and age at first breeding did not increase over time (see Figure S3), despite increasing densities that reached eastern levels by the end of the study period.

We found that juvenile survival of storks was more variable across years than survival in older age classes and exhibited synchrony over a large geographic range, while survival of older storks showed no synchrony. This pattern aligns with findings from other species, where juvenile survival is often more temporally variable than adult survival (e.g. Souchay et al., 2013). Greater sensitivity of juveniles to environmental conditions, resulting in increased temporal variability relative to adults and enhanced synchrony between populations, has previously been documented in two stork populations (Schaub et al., 2005). Our analysis extends this earlier work by quantifying synchrony across the entire range of spatial units. The persistence of synchrony in juvenile survival over a large spatial range revealed by our analysis suggests that shared, large‐scale environmental factors play a dominant role to induce temporal variation in juvenile survival.

In conclusion, our study reveals substantial large‐scale spatial variation in key demographic parameters in a long‐lived species. These differences likely contribute to the observed spatially divergent population trends. Our comparison of alternative spatial structures shows that incorporating spatial autocorrelation in a way that reflects the species' ecology provides a useful compromise between ignoring spatial autocorrelation and relying on spatial smoothing approaches that treat all neighbouring locations equivalently. Future studies should integrate spatial variation in other vital rates (e.g. fecundity), use a finer spatial resolution, incorporate external environmental drivers as covariates or combine different demographic datasets through integrated population models (Schaub & Kéry, 2022), to achieve a more comprehensive understanding of drivers of population trends. More broadly, our findings emphasise the need to account for spatial heterogeneity in demographic rates when studying and managing species across large spatial areas.

## AUTHOR CONTRIBUTIONS

Matia Haïm Muller and Michael Schaub conceived the ideas and designed methodology; Christof Herrmann, Wolfgang Fiedler and Olaf Geiter collected and stored the data; Matia Haïm Muller, with the help of Fabian R. Ketwaroo, analysed the data; Matia Haïm Muller led the writing of the manuscript. All authors critically revised earlier drafts and gave final approval for publication.

## CONFLICT OF INTEREST STATEMENT

We declare no conflict of interest.

## STATEMENT ON INCLUSION

The heads of the three German ringing schemes that organised the data collection and management are co‐authors of the paper. Their involvement ensured the integration of local knowledge, expertise and perspectives, thereby fostering collaboration, the accurate interpretation of regional data and direct engagement with stakeholders in Germany.

## Supporting information


**Appendix S1.** Modelling details.

**Appendix S2.** Secondary results.

**Appendix S3.** Results from models M1 and M2.

**Appendix S4.** Simulation study to assess the estimation of dead‐recovery probabilities.

**Figure S1.** Box plots of parameter uncertainty expressed as the width of the 95% credible interval (CRI) under each model (M1, M2, M3).
**Figure S2.** Posterior means (bold lines) and 95% credible intervals (bands) of annual survival probability (4 age classes) across time (2000–2022) for each spatial unit (see Table 1 for unit codes) obtained from model M3. The figure is structured by flyway.
**Figure S3.** Posterior means (bold lines) and 95% credible interval (bands) of time‐dependent age at first breeding (2001–2023) for each spatial unit obtained from model M3. Asterisks in MV indicate estimates with R‐hat >1.1.
**Table S1.** Unit‐specific means and temporal variability (standard deviations, SD) of survival, age at first breeding, resighting and recovery probabilities obtained from model M3. Given are posterior means and 95% CRI in brackets.
**Table S2.** Posterior probability per spatial unit that the temporal range in survival (2000–2022) for one age class exceeds that of another (obtained from model M3).

## Data Availability

Data and code available from the vogelwarte.ch Open Repository and Archive https://doi.org/10.5281/zenodo.20185338 (Muller et al., 2026).
