# Supplementary material for: Assessment of large‐scale spatial variation in age‐specific survival and age at first breeding in a long‐lived species
Source: J Anim Ecol. 2026 Jun 5;95(7):1260–72. doi: 10.1111/1365-2656.70291 (PMC13322180; doi:10.1111/1365-2656.70291)
Supplement: Supplementary file 1 — Appendix S1. Modelling details. [file JANE-95-1260-s005.pdf]

## APPENDIX S1. MODELLING DETAILS

In this appendix we describe some details of models that were used.

### 1. Normal-Normal state-space model

Annual counts of the number of occupied white stork nests for each federal state (Bundesland) from 2000-2023 were obtained from the NABU Bundesarbeitsgruppe Weißstorchschutz (NABU Federal Working Group for White Stork Conservation; data sources listed at the end of this appendix). These state-level counts were then summed across the three migratory flyway categories (western, eastern, and mixed) according to the assignment of each state (spatial unit) as described in Section 2.1.

We analysed annual changes in the number of breeding pairs per flyway using a Normal–Normal state-space model (Schaub & Kéry, 2022), which separates biological variability from observation error. Let $x_{t,f}$ denote the latent (true) log-abundance per year $t$ and flyway $f$ and $y_{t,f}$ the observed log-count. For each flyway, the population size in the year 2000 (initial value) is assigned a weakly informative prior: its latent log-abundance is drawn from a Normal distribution centred on the observed log count in 2000 with a large standard deviation (sd = 10). Then, the model assumes a Gaussian random-walk process for population dynamics:

$$x_{t,f} \sim Normal\left(x_{t-1,f} + r_f, \sigma_f^{2\,(proc)}\right), \qquad \text{(eqn A1-1)}$$

and a Gaussian observation model,

$$y_{t,f} \sim Normal\left(x_{t,f}, \sigma_f^{2\,(obs)}\right). \qquad \text{(eqn A1-2)}$$

$r_f$ is the flyway-specific mean annual growth rate (i.e. population trend, on the log scale), $\sigma_f^{2\,(proc)}$ is the flyway-specific standard deviation of the temporal variability of the biological process, and $\sigma_f^{2\,(obs)}$ is the flyway-specific standard deviation of the observation process.

## 2. Dimension reduction of the multistate capture-recapture-recovery (MCRR) model

The m-array data are analysed with a multinomial likelihood for each release cohort $z$:

$$\boldsymbol{m}_{i,z} = (m_{i,z,1}, \dots, m_{i,z,T+1}) \sim Multinomial(\boldsymbol{\pi}_{i,z}, R_{i,z}), \qquad \text{(eqn A1-3)}$$

where vector $\boldsymbol{\pi}_{i,z} = (\pi_{i,z,1}, \dots, \pi_{i,z,T+1})$ contains elements which are functions of the survival, transition and resighting probabilities that we aim to estimate and that sum to one (i.e., $\sum_{t=1}^{T+1} \pi_{i,z,t} = 1$; see the main text for more explanations). Both $\boldsymbol{m}$ (the m-arrays) and $\boldsymbol{\pi}$ are three dimensional arrays with the first dimension being the spatial unit $i$, the second dimension (corresponding to the rows of the m-arrays) being the release cohort $z$ (of size $Z$, which is the total number of states x total number of years), and the third dimension (corresponding to the columns of the m-arrays) corresponds to the possible outcomes for each cohort, including all re-encounter state–year combinations plus the "never re-encountered" category (the size of the third dimension is thus $Z + 1$). The computational time for the multinomial distribution increases strongly with the number of elements, i.e. with $Z$. Therefore, the principle to reduce computation time is the reduction of the dimensions of $\boldsymbol{\pi}$ and of $\boldsymbol{m}$ without changing the likelihood. Below we describe in detail how we have done that for our model.

The first step in the dimension reduction was to remove unnecessary elements in $\boldsymbol{m}$. We removed (1) all elements in all columns of the m-arrays that belong to unobservable states (i.e., states 9 to 16, leaving only 8 columns of re-encounters for each year), (2) all elements in all rows of the m-arrays belonging to unobservable states (corresponding to observation probabilities that equal to zero) and (3) all elements in all rows of the m-arrays corresponding to states that can only transition into unobservable states (states 5 to 8, which can only transition to "long dead"). Then we merged elements in columns whose states differed only by age class. This can be done, because for each state and year of release, there is only one possible age class in which an individual can be recaptured in a given year. The probability of recapture in the other age classes is 0, even if the state in principle is observable. We thus finally had only elements in two columns of re-encounters for each year: alive (the four

states juvenile, 1y breeder, 2y breeder and ≥3y breeder) and dead (the four states 1y recently dead, 2y recently dead, 3y recently dead and ≥4y recently dead). This merging was possible because the age class at re-encounter could be determined from the known age class at release and the time elapsed between release and re-encounter. Fig. A1-1 shows the dimension reduction process in a simple m-array. Our original $\boldsymbol{m}$ had dimensions 12 × 368 × 369 (=1’629’504 elements), whereas the optimized $\boldsymbol{m}^*$ was reduced to 12 × 92 × 47 (=51’888 elements which is about 31 times less).

| | Y2S1 | Y2S2 | Y2S3 | Y2S4 | Y2S5 | Y3S1 | Y3S2 | Y3S3 | Y3S4 | Y3S5 | Never |
|---|---|---|---|---|---|---|---|---|---|---|---|
| Y1S1 | 0 | 12 | 0 | 3 | 0 | 0 | 0 | 9 | 1 | 0 | 28 |
| Y1S2 | 0 | 0 | 8 | 2 | 0 | 0 | 0 | 6 | 1 | 0 | 21 |
| Y1S3 | 0 | 0 | 7 | 2 | 0 | 0 | 0 | 4 | 0 | 0 | 16 |
| Y1S4 | 0 | 0 | 0 | 0 | 0 | 0 | 0 | 0 | 0 | 0 | 0 |
| Y1S5 | 0 | 0 | 0 | 0 | 0 | 0 | 0 | 0 | 0 | 0 | 0 |
| Y2S1 | 0 | 0 | 0 | 0 | 0 | 0 | 14 | 0 | 2 | 0 | 32 |
| Y2S2 | 0 | 0 | 0 | 0 | 0 | 0 | 0 | 11 | 0 | 0 | 28 |
| Y2S3 | 0 | 0 | 0 | 0 | 0 | 0 | 0 | 12 | 3 | 0 | 25 |
| Y2S4 | 0 | 0 | 0 | 0 | 0 | 0 | 0 | 0 | 0 | 0 | 7 |
| Y2S5 | 0 | 0 | 0 | 0 | 0 | 0 | 0 | 0 | 0 | 0 | 0 |

| | Y2A | Y2D | Y3A | Y3D | Never |
|---|---|---|---|---|---|
| Y1S1 | 12 | 3 | 9 | 1 | 28 |
| Y1S2 | 8 | 2 | 6 | 1 | 21 |
| Y1S3 | 7 | 2 | 4 | 0 | 16 |
| Y2S1 | 0 | 0 | 14 | 2 | 32 |
| Y2S2 | 0 | 0 | 11 | 0 | 28 |
| Y2S3 | 0 | 0 | 12 | 3 | 25 |

**FIGURE A1-1.** Example showing the dimension reduction of a simple m-array. To the left, there is the m-array before dimension reduction, right there is the m-array after the dimension reduction. The 5 states are the following: S1 = alive as juvenile, S2 = alive as 1y, S3 = alive as ≥2y, S4 = recently (i.e., within the last year) dead, S5 = long dead (unobservable state). Y1 refers to year 1, Y2 to year 2. In the non-dimension-reduced m-array, year and state are combined into a single label (e.g. Y1S1 for juveniles alive in year 1). Elements in columns and rows deleted during the dimension reduction are shown in orange, and elements of columns merged are shown in blue and green. Elements of columns and rows belonging to S5 were deleted because S5 is an unobservable state. Elements in rows belonging to S4 were deleted because S4 can only transition to S5 (long dead). Elements in columns belonging to S1, S2 and S3 were merged because they differed only by age. In the column labels of the optimised m-array, the A refers to alive (the three columns belonging to S1, S2 and S3 are

merged), and the D refers to dead (the previous S4 column). Both are always combined with the year label (e.g. Y2D refers to recently dead in year 2).

Elements of $\boldsymbol{\pi}$ that refer to transitions before the release of individuals have a value of 0, meaning they do not contribute to the likelihood. However, they are still involved in the computation when the multinomial distribution is applied. Therefore, in a second step, we adapted the computation of the multinomial distribution to include only those elements of $\boldsymbol{\pi}$ that occur after the release of the individuals. The finally obtained multinomial likelihood was constructed as:

$$\boldsymbol{m}^*_{i,z^*} = \left(m^*_{i,z^*,\boldsymbol{k}}, \dots, m^*_{i,z^*,T^*+1}\right) \sim Multinomial\left(\boldsymbol{\pi}^*_{i,z^*}, R^*_{i,z^*}\right), \qquad \text{(eqn A1-4)}$$

with $k$ being the index of the first column in the m-array corresponding to the year following the release of individuals from cohort $z^*$, and $\boldsymbol{\pi}^*_{i,z^*} = \left(\pi^*_{i,z^*,k}, \dots, \pi^*_{i,z^*,T^*+1}\right)$. This targeted computation eliminated unnecessary costly operations performed in the not reduced model and significantly reduced computational time without changing the likelihood.

To verify that the dimension reduced and not dimension reduced formulations are the same, $\boldsymbol{\pi}^*$ values from both formulations were compared using identical, randomly generated spatial-, age- and time-varying demographic rates and encounter parameters. All non-zero $\boldsymbol{\pi}^*$ values were identical. This demonstrates that the dimension reduced model is structurally equivalent to the not dimension reduced model, has the same likelihood and therefore produces identical parameter estimates as the non-dimension reduced model. Importantly, it offers significantly improved computational efficiency: based on 100 iterations, it runs 26.79 times faster than the non-dimension reduced model.

## 3. Description of the intrinsic conditional autoregressive (ICAR) model

An intrinsic conditional autoregressive (ICAR) model (Besag, 1974) is a commonly used spatial model that induces spatial autocorrelation among neighbouring spatial units. It assumes that the random effect associated with a given spatial unit is conditionally

dependent on the values of neighbouring units. For example, for our set of spatial random effects on survival

$$\boldsymbol{\epsilon}^{\varphi}_{a_\varphi} = \left(\epsilon^{\varphi}_{1,a_\varphi}, \dots, \epsilon^{\varphi}_{12,a_\varphi}\right), \qquad \text{(eqn A1-5)}$$

the ICAR model specifies that each element $\epsilon^{(\varphi)}_{i,a_\varphi}$ follows a conditional normal distribution given all other element $\epsilon^{\varphi}_{-i,a_\varphi}$:

$$\epsilon^{\varphi}_{i,a_\varphi} | \epsilon^{\varphi}_{-i,a_\varphi} \sim Normal\left(\frac{1}{n_i}\sum_{g\in\delta_i}\epsilon^{\varphi}_{g,a_\varphi}, \frac{1}{n_i\tau^{\varphi}_{a_\varphi}}\right), \qquad \text{(eqn A1-6)}$$

where $\delta_i$ is the set of neighbours of spatial unit $i$, $n_i = |\delta_i|$ is the number of neighbours of spatial unit $i$ and $\tau^{\varphi}_{a_\varphi}$ is the precision parameter that controls the overall strength of spatial smoothing.

This formulation means that each random spatial effect tends to be similar to the average of its neighbours, thereby imposing positive spatial autocorrelation. The joint distribution of $\epsilon^{(\varphi)}_{i,a_\varphi}$ is improper (the precision matrix is singular), which is why a sum to zero constraint,

$$\sum_i \epsilon^{\varphi}_{i,a_\varphi} = 0, \qquad \text{(eqn A1-7)}$$

is imposed to ensure model identifiability.

In practice, the ICAR model corresponds to a multivariate normal distribution with mean zero:

$$\boldsymbol{\epsilon}^{\varphi}_{a_\varphi} \sim MVN\left(\mathbf{0}, \left[\tau^{\varphi}_{a_\varphi}(D^{\varphi} - \mathrm{W}^{\varphi})\right]^{-1}\right), \qquad \text{(eqn A1-8)}$$

where $\mathrm{W}^{(\varphi)}$ is the adjacency matrix, which defines which spatial units are neighbours (for example, in formulation M3, $\mathrm{W}^{\varphi}_{i,g} = 1$ if spatial units $i$ and $g$ share a border and belong to the same flyway area, 0 otherwise), and $D^{\varphi}$ is a diagonal matrix with entries $n_i$.

Although the above formulation is presented for survival, the same ICAR structure was applied to recruitment (with the same adjacency matrix as survival) and to dead-recovery

probabilities (which used a different adjacency matrix; see below), each parameter having its own precision.

## 4. Calculating the probability that the mean of a spatial unit is higher than the overall mean

To assess the support for spatial differences in survival, age at first breeding and natal emigration, we computed the posterior probability that the posterior mean of each spatial unit was higher than the posterior of the overall mean. This was achieved by calculating the proportion of posterior samples in which the mean of a given spatial unit was higher than the overall mean. We performed this computation separately for each age class in survival, as well as for age at first breeding and natal emigration.

## 5. Calculating the probability that the spatial range in survival for one age class exceeds that of another (pairwise)

For each MCMC iteration, we calculated for each age class the spatial range of survival as the difference between the maximum and minimum unit-specific mean survival. This yielded a posterior distribution of the spatial range of survival for each age class. We then computed the probabilities that the range for one age class exceeded that for another age class as the proportion of MCMC iterations where this condition held.

## 6. Calculating the probability that the temporal range in survival for one age class exceeds that of another for each spatial unit

For each MCMC iteration, we calculated for each spatial unit and age class the temporal range in survival as the difference between the maximum and minimum annual survival values. We then computed the posterior probability that the temporal range for one age class

exceeds that for another age class as a proportion of the MCMC iterations in which the temporal range of one age class was larger than that of the other.

## 7. Computation of the probability of positive correlation in temporal variability of survival between spatial units

To infer synchrony in survival, we assessed the strength of correlations in temporal variability (i.e., time series) of survival across spatial units. We computed, for each age class and MCMC sample, the pairwise correlation between annual fluctuations in survival $\zeta^{(\varphi)}_{i,a_\varphi,1:T}$ from two spatial units $i$ and $i'$ ($cor_{i,i',a_\varphi}$). From these posterior distributions we calculated the proportion of posterior samples where $cor_{i,i',a_\varphi} > 0$, which represents the probability of a positive correlation between the spatial units $i$ and $i'$.

## 8. Computation of overall mean survival and overall mean recruitment

In model M1, spatial units are modelled independently from each other and thus no overall mean parameter is explicitly defined. We therefore calculated the overall mean survival $\Phi_{a_\varphi}$ and an overall mean recruitment $\Gamma_{a_\gamma}$ as averages across spatial units:

$$\Phi_{a_\varphi} = logit^{-1}\left(\frac{1}{I}\sum_{i=1}^{I}\mu^{\varphi}_{i,a_\varphi}\right) \qquad \text{(eqn A1-9)}$$

$$\Gamma_{a_\gamma} = logit^{-1}\left(\frac{1}{I}\sum_{i=1}^{I}\mu^{\gamma}_{i,a_\gamma}\right), \qquad \text{(eqn A1-10)}$$

where $I$ is the number of spatial units and $\mu^{\varphi}_{i,a_\varphi}$ and $\mu^{\gamma}_{i,a_\gamma}$ are the spatial unit-specific across-years means of age-dependent survival and recruitment, respectively.

From models M2 and M3 we simply back-transformed the logit-scale overall means to get the overall means:

$$\Phi_{a_\varphi} = logit^{-1}\left(\mu^{\varphi}_{a_\varphi}\right) \qquad \text{(eqn A1-11)}$$

$$\Gamma_{a_\gamma} = logit^{-1}\left(\mu^{\gamma}_{a_\gamma}\right). \quad \text{(eqn A1-12)}$$

## 9. Computation of the overall mean age at first breeding

To obtain the overall mean age at first breeding, we first calculated the spatially averaged probability $\mathrm{K}_{a_\gamma}$ that a new-born will start breeding at each age class $a_\gamma = 1, \dots, 5$, conditional on its survival until that age class:

$$\mathrm{K}_{a_\gamma} = \begin{cases} \Gamma_{a_\gamma}, & if\, a_\gamma = 1 \\ \Gamma_{a_\gamma}\left(1 - \sum_{j=1}^{a_\gamma - 1} \mathrm{K}_j\right), & if\ 1 < a_\gamma < 5. \\ 1 - \sum_{j=1}^{4} \mathrm{K}_j, & if\ a_\gamma = 5 \end{cases} \quad \text{(eqn A1-13)}$$

The overall mean age at first breeding was then obtained as

$$\bar{A} = \sum_{j=1}^{5} j\, \mathrm{K}_j. \quad \text{(eqn A1-14)}$$

## 10. Temporal variation in the age at first breeding

To calculate year-specific age at first breeding in each spatial unit, we first calculated time-dependent absolute probability of recruitment $\varrho_{i,a_\gamma,t}$ (i.e., the probability that an individual breeds for the first time at age $a_\gamma$ (from 1 to 5) in year $t$) for each spatial unit and age class from the estimated recruitment probabilities $\gamma$:

$$\varrho_{i,a_\gamma,t} = \begin{cases} \gamma_{i,a_\gamma,t}, & if\, a_\gamma = 1 \\ \gamma_{i,a_\gamma,t}\left(1 - \sum_{j=1}^{a_\gamma - 1} \varrho_{i,j,t}\right), & if\ 1 < a_\gamma < 5. \\ 1 - \sum_{j=1}^{4} \varrho_{i,j,t}, & if\ a_\gamma = 5 \end{cases} \quad \text{(eqn A1-15)}$$

The age at first breeding in year $t$ and unit $i$ ($Z_{i,t}$) was then obtained as follows:

$$Z_{i,t} = \sum_{j=1}^{5} j\, \varrho_{i,j,t}. \quad \text{(eqn A1-16)}$$

### 11. Resighting probability

Immediate trap-response effects, whereby the probability of resighting an individual depends on whether it was seen the previous year, are common in capture–recapture studies and can bias parameter estimates if unaccounted for (Pradel, 1993). To assess whether trap-response was present in our data, we performed a goodness of fit test using the adult resighting data in the capture-recapture matrix format with the R package R2ucare (Gimenez et al., 2018). Specifically, we used Test 2.CT, which is sensitive to immediate trap-response. This test compares the frequency of recaptures at occasion $t+1$ between individuals that were and that were not captured at occasion $t$. Test 2.CT was highly significant in most spatial units, with more individuals resighted than expected in the year following a previous detection (Table A1-1), indicating immediate trap-happiness (Pradel, 1993).

**TABLE A1-1.** Results of Test 2.CT in each spatial unit. Negative values in the signed statistic indicate immediate trap-happiness, while positive values indicate immediate trap-shyness. df = degrees of freedom. See table 1 for the abbreviations of region codes (spatial units).

| **Spatial unit** | **BW** | **RP** | **BY** | **HE** | **NW** | **TH** | **NI** | **SH** | **ST** | **SN** | **MV** | **BB** |
|---|---|---|---|---|---|---|---|---|---|---|---|---|
| $\chi^2$ Statistic | 490.98 | 51.60 | 25.42 | 175.64 | 40.67 | 0.00 | 116.69 | 68.28 | 71.37 | 24.63 | 27.17 | 90.39 |
| df | 21 | 11 | 12 | 19 | 13 | 2 | 15 | 17 | 21 | 21 | 15 | 21 |
| Signed Statistic | -17.02 | -4.19 | -3.18 | -9.96 | -2.77 | 0.00 | -7.37 | -5.57 | -6.78 | -3.99 | -3.04 | -8.47 |
| p-value | **0.00** | **0.00** | **0.01** | **0.00** | **0.00** | 1.00 | **0.00** | **0.00** | **0.00** | 0.26 | 0.06 | **0.00** |

As we considered only live resightings of breeding individuals, we did not include age-dependence in resighting probability. However, we accounted for temporal variability, since monitoring effort is rarely constant over more than two decades. Furthermore, to allow for

potential differences in monitoring procedures between spatial units, each unit was modelled independently.

We therefore modelled resighting probability as a function of year $t$, spatial unit $i$ and whether the individual was or was not seen in year $t$-1 (variable $x$ in the following equation):

$$\mathrm{logit}\left(p_{x,i,t}\right) = \mu^{p}_{x,i} + \zeta^{p}_{x,i,t}, \qquad \text{(eqn A1-17)}$$

where $\mu^{p}_{x,i}$ is the trap- and unit-dependent mean, and $\zeta^{p}_{x,i,t}$ is an independent temporal random effect, defined as $\zeta^{p}_{x,i,t} \sim Normal(0, \sigma^{p}_{x,i})$, where $\sigma^{p}_{x,i}$ is the standard deviation of temporal variability, treated as a model parameter with its own prior.

## 12. Recovery probability

One-year-old white storks do not consistently return to the breeding grounds from their wintering areas during the breeding season (Van den Bossche et al., 2002), and 2- and 3-year-old individuals typically arrive later on the breeding grounds than older individuals (Belabed et al., 2019; Vergara et al., 2007). Due to this age-specific behaviour and the likely different recovery probabilities between breeding (i.e., Germany) and non-breeding (i.e., outside Germany) areas, we assumed that dead recovery probability was age-dependent, with four age classes ($a_r = 1 \ldots 4$). Moreover, given the large geographic extent of our study area (Germany) and regional differences in wintering areas, we assumed recovery probability to vary among spatial units from which storks originate.

The joint estimation of age-specific survival and age-specific recovery probabilities generally requires releases of individuals in all age classes considered (Anderson et al., 1985). However, only nestlings were ringed, and resightings were limited to breeding individuals. As a result, we had very few re-encounters of 1-year-old individuals and 2-year-old individuals, making it statistically infeasible to estimate four distinct age-dependent recovery probabilities per spatial unit.

We therefore developed a parameterization that allowed us to retain age-dependency in recovery probabilities without overparameterizing the model. We assumed that differences across age classes in recovery probability were driven solely by the location of the recovery (binary: inside or outside Germany) rather than by differences in mortality causes, which are generally similar across ages in storks (Schaub & Pradel, 2004). We therefore defined two spatially varying but age-independent parameters per spatial unit: $rb_i$ and $rnb_i$, representing the probabilities that a stork ringed in unit $i$ is being found dead in Germany and outside Germany, respectively. These parameters were shared across all age classes. Thus, even if we estimated four age-dependent recovery probabilities per spatial unit, their dependence on shared parameters meant we effectively estimated only two recovery-related parameters per spatial unit that are identifiable. The age-dependent probability of dead recovery $r_{i,a_r}$ was then calculated for each spatial unit $i$ and age class $a$ as:

$$r_{i,a_r} = prop_{i,a_r} * rnb_i \ + \left(1 - prop_{i,a_r}\right) * rb_i, \qquad \text{(eqn A1-18)}$$

where $prop_{i,a}$ is the proportion of birds from spatial unit *i* of age class $a$ recovered outside Germany, estimated from a binomial distribution from our data:

$$O_{i,a_r} \sim Binomial\left(prop_{i,a_r}, R_{i,a_r}\right), \qquad \text{(eqn A1-19)}$$

with $O_{i,a_r}$ being the number of individuals ringed in spatial unit $i$ and recovered at age class $a$ outside Germany, and $R_{i,a_r}$ being the age-specific total number of individuals ringed in unit $i$ that were recovered.

$rb_i$ was modelled with spatial autocorrelation:

$$\boldsymbol{\epsilon}^{rb} \sim ICAR\ (\mathrm{W}^{rb}, \tau^{rb}) \qquad \text{(eqn A1-20)}$$

$$rb_i = \mu^{rb} + \epsilon_i^{rb}, \qquad \text{(eqn A1-21)}$$

where $\boldsymbol{\epsilon}^{rb} = \left(\epsilon_{i=1}^{rb}, \ldots, \epsilon_{i=12}^{rb}\right)$, $\tau^{rb}$ is the precision, $\mathrm{W}^{rb}$ is the adjacency matrix, which considers as neighbours all spatial units sharing a direct border (i.e., the same adjacency structure as in model M2 for survival and recruitment), and $\mu^{rb}$ is the overall mean probability (at the logit-scale) that a stork is being found dead outside Germany.

$rnb_i$ was modelled without spatial autocorrelation, assuming spatial independence between spatial units.

To validate our approach, we conducted a simulation study to evaluate the accuracy with which our model could identify age-dependent recovery probabilities, and to determine whether it outperformed a simpler model that assumed a constant recovery probability across ages. The simulation results are shown in Appendix S4. They confirmed the reliability and improved performance of our method.

## 13. Priors

The table below show the prior distributions used for the parameters in the models.

**TABLE A1-2.** List of the prior distributions used in the models. Subscript numbers indicate age class. When $i$ is used as a subscript, the same prior was applied across all spatial units. Some priors are specific to particular models; the corresponding column therefore indicates in which model(s) each prior was used.

| Parameter | Model | Prior distribution |
|---|---|---|
| $\mu_{i,1}^{\varphi}$ | M1 | $Normal\ (\mu = 0, \sigma = 3)$ |
| $\mu_{i,2}^{\varphi}$ | M1 | $Normal\ (\mu = 0, \sigma = 3)$ |
| $\mu_{i,3}^{\varphi}$ | M1 | $Normal\ (\mu = 0, \sigma = 3)$ |
| $\mu_{i,4}^{\varphi}$ | M1 | $Normal\ (\mu = 0, \sigma = 3)$ |
| $\mu_{1}^{\varphi}$ | M2, M3 | $Normal\ (\mu = 0, \sigma = 3)$ |
| $\mu_{2}^{\varphi}$ | M2, M3 | $Normal\ (\mu = 0, \sigma = 3)$ |
| $\mu_{3}^{\varphi}$ | M2, M3 | $Normal\ (\mu = 0, \sigma = 3)$ |
| $\mu_{4}^{\varphi}$ | M2, M3 | $Normal\ (\mu = 0, \sigma = 3)$ |
| $\mu_{i,1}^{\gamma}$ | M1 | $Normal\ (\mu = 0, \sigma = 3)$ |
| $\mu_{i,2}^{\gamma}$ | M1 | $Normal\ (\mu = 0, \sigma = 3)$ |

| | | |
|---|---|---|
| $\mu_{i,3}^{\gamma}$ | M1 | $Normal\ (\mu = 0, \sigma = 3)$ |
| $\mu_{i,4}^{\gamma}$ | M1 | $Normal\ (\mu = 0, \sigma = 3)$ |
| $\mu_{1}^{\gamma}$ | M2, M3 | $Normal\ (\mu = 0, \sigma = 3)$ |
| $\mu_{2}^{\gamma}$ | M2, M3 | $Normal\ (\mu = 0, \sigma = 3)$ |
| $\mu_{3}^{\gamma}$ | M2, M3 | $Normal\ (\mu = 0, \sigma = 3)$ |
| $\mu_{4}^{\gamma}$ | M2, M3 | $Normal\ (\mu = 0, \sigma = 3)$ |
| $\mu_{1}^{\eta}$ | All | $Normal\ (\mu = 0, \sigma = 3)$ |
| $\mu_{2}^{\eta}$ | All | $Normal\ (\mu = 0, \sigma = 3)$ |
| $\mu^{rb}$ | All | $Normal\ (\mu = 0, \sigma = 3)$ |
| $\mu_{seen\ the\ year\ before,i}^{(p)}$ | All | $Normal\ (\mu = 0, \sigma = 3)$ |
| $\mu_{not\ seen\ the\ year\ before,i}^{(p)}$ | All | $Normal\ (\mu = 0, \sigma = 3)$ |
| $\sigma_{i,2}^{\varphi}$ | All | $Uniform\ (0, 10)$ |
| $\sigma_{i,2}^{\varphi}$ | All | $Uniform\ (0, 10)$ |
| $\sigma_{i,3}^{\varphi}$ | All | $Uniform\ (0, 10)$ |
| $\sigma_{i,4}^{\varphi}$ | All | $Uniform\ (0, 10)$ |
| $\sigma_{i,1}^{\gamma}$ | All | $Uniform\ (0, 10)$ |
| $\sigma_{i,2}^{\gamma}$ | All | $Uniform\ (0, 10)$ |
| $\sigma_{i,2}^{\gamma}$ | All | $Uniform\ (0, 10)$ |
| $\sigma_{i,2}^{\gamma}$ | All | $Uniform\ (0, 10)$ |
| $\sigma_{1}^{\eta}$ | All | $Uniform\ (0, 10)$ |
| $\sigma_{2}^{\eta}$ | All | $Uniform\ (0, 10)$ |
| $\sigma_{seen\ the\ year\ before,i}^{p}$ | All | $Uniform\ (0, 10)$ |
| $\sigma_{not\ seen\ the\ year\ before,i}^{p}$ | All | $Uniform\ (0, 10)$ |
| $rnb_i$ | All | $Uniform\ (0, 1)$ |
| $prop_{i,1}$ | All | $Uniform\ (0, 1)$ |
| $prop_{i,2}$ | All | $Uniform\ (0, 1)$ |
| $prop_{i,3}$ | All | $Uniform\ (0, 1)$ |

| | | |
|---|---|---|
| $prop_{i,4}$ | All | $Uniform\ (0, 1)$ |
| $\tau_1^{\varphi}$ | M2, M3 | $Gamma\ (0.001, 0.001)$ |
| $\tau_2^{\varphi}$ | M2, M3 | $Gamma\ (0.001, 0.001)$ |
| $\tau_3^{\varphi}$ | M2, M3 | $Gamma\ (0.001, 0.001)$ |
| $\tau_4^{\varphi}$ | M2, M3 | $Gamma\ (0.001, 0.001)$ |
| $\tau_1^{\gamma}$ | M2, M3 | $Gamma\ (0.001, 0.001)$ |
| $\tau_2^{\gamma}$ | M2, M3 | $Gamma\ (0.001, 0.001)$ |
| $\tau_3^{\gamma}$ | M2, M3 | $Gamma\ (0.001, 0.001)$ |
| $\tau_4^{\gamma}$ | M2, M3 | $Gamma\ (0.001, 0.001)$ |
| $\tau^{rb}$ | All | $Gamma\ (0.001, 0.001)$ |

## References


Anderson, D. R., Burnham, K. P., & White, G. C. (1985). Problems in Estimating Age-Specific Survival Rates from Recovery Data of Birds Ringed as Young. *Journal of Animal Ecology*, *54*(1), 89–98. https://doi.org/10.2307/4622

Belabed, B.-E., Athamnia, M., Touati, L., Samraoui, F., Boucheker, A., & Samraoui, B. (2019). The early bird catches the worm: Age-specific arrival time influences reproductive performance in the White Stork *Ciconia ciconia*. *Bird Study*, *66*(1), 121–129. https://doi.org/10.1080/00063657.2019.1618240

Besag, J. (1974). Spatial Interaction and the Statistical Analysis of Lattice Systems. *Journal of the Royal Statistical Society Series B: Statistical Methodology*, *36*(2), 192–225. https://doi.org/10.1111/j.2517-6161.1974.tb00999.x

Gimenez, O., Lebreton, J.-D., Choquet, R., & Pradel, R. (2018). *R2ucare: An R package to perform goodness-of-fit tests for capture-recapture models* (p. 192468). bioRxiv. https://doi.org/10.1101/192468

Pradel, R. (1993). Flexibility in Survival Analysis from Recapture Data: Handling Trap-Dependence. In J.-D. L. & P. M. North (Ed.), *Marked individuals in the study of bird populations* (pp. 29–37). Birkhäuser Verlag. https://hal.science/hal-02928241

Schaub, M., & Kéry, M. (2022). *Integrated Population Models: Theory and Ecological Applications with R and JAGS*. Elsevier Science.

Schaub, M., & Pradel, R. (2004). Assessing the Relative Importance of Different Sources of Mortality from Recoveries of Marked Animals. *Ecology*, *85*(4), 930–938. https://doi.org/10.1890/03-0012

Van den Bossche, W., Berthold, P., Kaatz, M., Nowak, E., & Querner, U. (2002). *Eastern European White Stork Populations: Migration Studies and Elaboration of Conservation Measures* (German Federal Agency for Nature Conservation).

Vergara, P., I. Aguirre, J., & Fernández-Cruz, M. (2007). Arrival date, age and breeding success in white stork Ciconia ciconia. *Journal of Avian Biology*, *38*(5), 573–579. https://doi.org/10.1111/j.2007.0908-8857.03983.x

**<u>Data sources</u>**

Weißstorchschutz, N. D. B. (2001). Mitteilungsblatt 93/2001 der BAG Weißstorchschutz. NABU, Bundesfachausschuss Ornithologie, Bundesarbeitsgruppe Weißstorchschutz.

Weißstorchschutz, N. D. B. (2002). Mitteilungsblatt 94/2002 der BAG Weißstorchschutz. NABU, Bundesfachausschuss Ornithologie, Bundesarbeitsgruppe Weißstorchschutz.

Weißstorchschutz, N. D. B. (2003). Mitteilungsblatt 95/2003 der BAG Weißstorchschutz. NABU, Bundesfachausschuss Ornithologie, Bundesarbeitsgruppe Weißstorchschutz.

Weißstorchschutz, N. D. B. (2004). Mitteilungsblatt 96/2004 der BAG Weißstorchschutz. NABU, Bundesfachausschuss Ornithologie, Bundesarbeitsgruppe Weißstorchschutz.

Weißstorchschutz, N. D. B. (2005). Mitteilungsblatt 97/2005 der BAG Weißstorchschutz. NABU, Bundesfachausschuss Ornithologie, Bundesarbeitsgruppe Weißstorchschutz.

Weißstorchschutz, N. D. B. (2006). Mitteilungsblatt 98/2006 der BAG Weißstorchschutz. NABU, Bundesfachausschuss Ornithologie, Bundesarbeitsgruppe Weißstorchschutz.

Weißstorchschutz, N. D. B. (2007). Mitteilungsblatt 99/2007 der BAG Weißstorchschutz. NABU, Bundesfachausschuss Ornithologie, Bundesarbeitsgruppe Weißstorchschutz.

Weißstorchschutz, N. D. B. (2008). Mitteilungsblatt 100/2008 der BAG Weißstorchschutz. NABU, Bundesfachausschuss Ornithologie, Bundesarbeitsgruppe Weißstorchschutz.

Weißstorchschutz, N. D. B. (2009). Mitteilungsblatt 101/2009 der BAG Weißstorchschutz. NABU, Bundesfachausschuss Ornithologie, Bundesarbeitsgruppe Weißstorchschutz.

Weißstorchschutz, N. D. B. (2010). Mitteilungsblatt 102/2010 der BAG Weißstorchschutz. NABU, Bundesfachausschuss Ornithologie, Bundesarbeitsgruppe Weißstorchschutz.

Weißstorchschutz, N. D. B. (2011). Mitteilungsblatt 103/2011 der BAG Weißstorchschutz. NABU, Bundesfachausschuss Ornithologie, Bundesarbeitsgruppe Weißstorchschutz.

Weißstorchschutz, N. D. B. (2012). Mitteilungsblatt 104/2012 der BAG Weißstorchschutz. NABU, Bundesfachausschuss Ornithologie, Bundesarbeitsgruppe Weißstorchschutz.

Weißstorchschutz, N. D. B. (2013). Mitteilungsblatt 105/2013 der BAG Weißstorchschutz. NABU, Bundesfachausschuss Ornithologie, Bundesarbeitsgruppe Weißstorchschutz.

Weißstorchschutz, N. D. B. (2014). Mitteilungsblatt 106/2014 der BAG Weißstorchschutz. NABU, Bundesfachausschuss Ornithologie, Bundesarbeitsgruppe Weißstorchschutz.

Weißstorchschutz, N. D. B. (2015). Mitteilungsblatt 107/2015 der BAG Weißstorchschutz. NABU, Bundesfachausschuss Ornithologie, Bundesarbeitsgruppe Weißstorchschutz.

Weißstorchschutz, N. D. B. (2016). Mitteilungsblatt 108/2016 der BAG Weißstorchschutz. NABU, Bundesfachausschuss Ornithologie, Bundesarbeitsgruppe Weißstorchschutz.

Weißstorchschutz, N. D. B. (2017). Mitteilungsblatt 109/2017 der BAG Weißstorchschutz. NABU, Bundesfachausschuss Ornithologie, Bundesarbeitsgruppe Weißstorchschutz.

Weißstorchschutz, N. D. B. (2018). Mitteilungsblatt 110/2018 der BAG Weißstorchschutz. NABU, Bundesfachausschuss Ornithologie, Bundesarbeitsgruppe Weißstorchschutz.

Weißstorchschutz, N. D. B. (2019). Mitteilungsblatt 111/2019 der BAG Weißstorchschutz. NABU, Bundesfachausschuss Ornithologie, Bundesarbeitsgruppe Weißstorchschutz.

Weißstorchschutz, N. D. B. (2020). Mitteilungsblatt 112/2020 der BAG Weißstorchschutz. NABU, Bundesfachausschuss Ornithologie, Bundesarbeitsgruppe Weißstorchschutz.

Weißstorchschutz, N. D. B. (2021). Mitteilungsblatt 113/2021 der BAG Weißstorchschutz. NABU, Bundesfachausschuss Ornithologie, Bundesarbeitsgruppe Weißstorchschutz.

Weißstorchschutz, N. D. B. (2022). Mitteilungsblatt 114/2022 der BAG Weißstorchschutz. NABU, Bundesfachausschuss Ornithologie, Bundesarbeitsgruppe Weißstorchschutz.

Weißstorchschutz, N. D. B. (2023). Mitteilungsblatt 115/2023 der BAG Weißstorchschutz. NABU, Bundesfachausschuss Ornithologie, Bundesarbeitsgruppe Weißstorchschutz.

Weißstorchschutz, N. D. B. (2024). Mitteilungsblatt 116/2024 der BAG Weißstorchschutz. NABU, Bundesfachausschuss Ornithologie, Bundesarbeitsgruppe Weißstorchschutz.
