# Supplementary material for: Assessment of large‐scale spatial variation in age‐specific survival and age at first breeding in a long‐lived species
Source: J Anim Ecol. 2026 Jun 5;95(7):1260–72. doi: 10.1111/1365-2656.70291 (PMC13322180; doi:10.1111/1365-2656.70291)
Supplement: Supplementary file 2 — Appendix S2. Secondary results. [file JANE-95-1260-s001.pdf]

APPENDIX S2 – SECONDARY RESULTS

Here we present estimates of the observation parameters as obtained from the best fitting model M3.

1. **Resighting probability**

The probability of resighting was importantly higher if an individual had been seen in the previous year than if it had not (trap-happiness; Fig. A2-1, Table A2-1). One possible explanation for the trap-happy effect is observer behaviour, as places where a ringed individual was seen in the previous year may be more likely to be checked again the following year.

The mean resighting probabilities varied across spatial units, but no clear spatial structure emerged (Table A2-1). The temporal variability in resighting probability was greater in western Germany than in the east (Fig. A2-1, Table A2-1).

Together, these results show the importance of accounting for immediate trap-response and spatio-temporal variation.

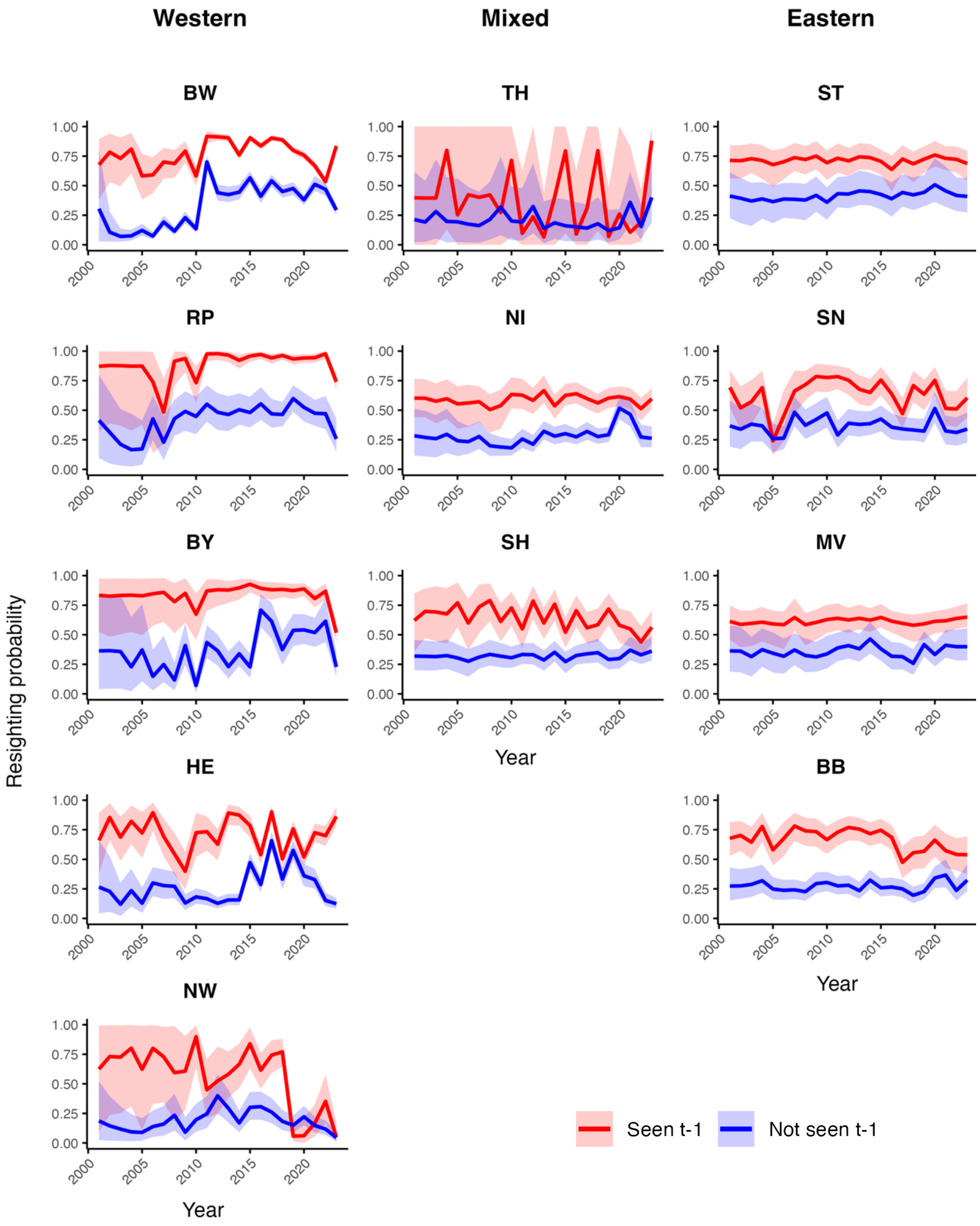

FIGURE A2-1. Posterior means (bold lines) and 95% credible interval (bands) of resighting probabilities in year t as a function of whether the individual was seen the previous year ($t-1$) for each spatial unit.

TABLE A2-1. Unit-specific posterior means and 95% credible intervals (brackets) of parameters associated with resighting and dead recovery probabilities. The flyway associated with each unit is given. Standard deviation is abbreviated as SD. The grey layers have no meaning but should increase the readability of the table.

Flyway	Western					Mixed			Eastern			
Spatial unit	BW	RP	BY	HE	NW	TH	NI	SH	ST	SN	MV	BB
Mean resighting probability if seen the previous year	0.787 (0.717-0.843)	0.934 (0.872-0.973)	0.858 (0.795-0.914)	0.741 (0.650-0.821)	0.572 (0.346-0.802)	0.289 (0.008-0.892)	0.589 (0.532-0.641)	0.658 (0.575-0.746)	0.719 (0.661-0.775)	0.641 (0.558-0.718)	0.615 (0.556-0.668)	0.673 (0.613-0.733)
Mean resighting probability if not seen the previous year	0.288 (0.191-0.397)	0.400 (0.280-0.505)	0.336 (0.216-0.466)	0.239 (0.168-0.319)	0.164 (0.102-0.236)	0.184 (0.086-0.325)	0.274 (0.220-0.334)	0.318 (0.265-0.373)	0.413 (0.335-0.493)	0.364 (0.303-0.426)	0.357 (0.292-0.425)	0.270 (0.227-0.318)
SD resighting probability if seen the previous year	0.836 (0.586-1.224)	1.516 (0.857-2.552)	0.840 (0.508-1.368)	0.941 (0.618-1.433)	2.109 (1.283-3.475)	5.720 (1.179-9.756)	0.308 (0.038-0.648)	0.647 (0.322-1.114)	0.298 (0.023-0.674)	0.738 (0.479-1.100)	0.225 (0.012-0.622)	0.539 (0.314-0.837)
SD resighting probability if not seen the previous year	1.169 (0.831-1.645)	0.817 (0.312-1.484)	1.135 (0.715-1.748)	0.893 (0.617-1.298)	0.853 (0.484-1.384)	0.884 (0.289-1.753)	0.494 (0.312-0.755)	0.233 (0.026-0.507)	0.306 (0.022-0.770)	0.410 (0.215-0.663)	0.361 (0.076-0.668)	0.321 (0.145-0.536)
1y recovery probability	0.094 (0.086-0.104)	0.109 (0.095-0.125)	0.118 (0.105-0.132)	0.106 (0.093-0.120)	0.080 (0.068-0.093)	0.073 (0.057-0.092)	0.059 (0.053-0.065)	0.079 (0.070-0.090)	0.055 (0.048-0.062)	0.048 (0.043-0.054)	0.047 (0.041-0.053)	0.034 (0.030-0.039)

Flyway	Western					Mixed			Eastern			
Spatial unit	BW	RP	BY	HE	NW	TH	NI	SH	ST	SN	MV	BB
2y recovery probability	0.098 (0.091-0.107)	0.107 (0.089-0.122)	0.115 (0.091-0.139)	0.104 (0.082-0.124)	0.088 (0.071-0.113)	0.074 (0.047-0.112)	0.056 (0.043-0.066)	0.079 (0.062-0.096)	0.046 (0.030-0.061)	0.041 (0.028-0.052)	0.025 (0.012-0.046)	0.023 (0.014-0.033)
3y recovery probability	0.100 (0.085-0.113)	0.111 (0.088-0.136)	0.110 (0.076-0.139)	0.110 (0.086-0.136)	0.073 (0.042-0.096)	0.076 (0.053-0.103)	0.060 (0.051-0.070)	0.076 (0.057-0.094)	0.038 (0.020-0.057)	0.051 (0.042-0.061)	0.041 (0.029-0.053)	0.025 (0.015-0.036)
≥4y recovery probability	0.104 (0.087-0.126)	0.116 (0.092-0.148)	0.121 (0.101-0.145)	0.110 (0.094-0.133)	0.100 (0.077-0.131)	0.070 (0.036-0.105)	0.064 (0.055-0.076)	0.080 (0.069-0.095)	0.063 (0.052-0.077)	0.056 (0.047-0.066)	0.051 (0.043-0.059)	0.038 (0.032-0.046)
Recovery probability in Germany	0.119 (0.062-0.178)	0.122 (0.062-0.192)	0.122 (0.062-0.171)	0.116 (0.063-0.175)	0.128 (0.063-0.195)	0.077 (0.047-0.110)	0.074 (0.049-0.100)	0.068 (0.027-0.116)	0.078 (0.056-0.099)	0.064 (0.046-0.078)	0.059 (0.045-0.070)	0.053 (0.037-0.066)
Recovery probability outside Germany	0.074 (0.014-0.137)	0.095 (0.016-0.177)	0.108 (0.005-0.272)	0.096 (0.021-0.168)	0.051 (0.005-0.103)	0.067 (0.005-0.157)	0.041 (0.006-0.075)	0.100 (0.012-0.190)	0.020 (0.001-0.054)	0.020 (0.001-0.055)	0.014 (0.001-0.046)	0.011 (0.000-0.029)

2. Dead recovery probability

The recovery probabilities inside and outside of Germany were both lower in units belonging to the eastern flyway compared to units belonging to the western flyway (Table A2-1). In units belonging to the eastern flyway, 1y and 2y individuals had the lowest recovery probabilities, whereas adults had the highest (Table A2-1). This is consistent with our expectations: in the eastern flyway, younger birds (1y and 2y) are less likely to return to Germany in the breeding period, resulting in a longer time spent in areas such as the Middle East or East Africa, where dead recovery probabilities are lower.

Previous studies of spatial variation in survival have generally not explicitly considered spatial differences in the probability of dead recoveries, even when recoveries originated from an entire continent (e.g., Schirmer et al., 2023). According to our estimates, recovery probabilities are likely to vary even within a single country. Although this was not the main focus of our study, our results highlight the importance of accounting for spatial variation in both resighting and dead recovery probabilities when estimating demographic parameters over large areas.

References

Schirmer, S., Korner-Nievergelt, F., von Rönn, J. A. C., & Liebscher, V. (2023). Estimating survival in continuous space from mark-dead-recovery data—Towards a continuous version of the multinomial dead recovery model. *Journal of Theoretical Biology*, *574*, 111625. https://doi.org/10.1016/j.jtbi.2023.111625
