# Supplementary material for: Assessment of large‐scale spatial variation in age‐specific survival and age at first breeding in a long‐lived species
Source: J Anim Ecol. 2026 Jun 5;95(7):1260–72. doi: 10.1111/1365-2656.70291 (PMC13322180; doi:10.1111/1365-2656.70291)
Supplement: Supplementary file 3 — Appendix S3. Results from models M1 and M2. [file JANE-95-1260-s003.pdf]

APPENDIX S3 – RESULTS FROM MODELS M1 AND M2

1. Results from model M1 (independent)

1.1 Survival

The overall arithmetic mean annual survival probability across spatial units and years was 0.384 (95% CRI: 0.355–0.412) for juveniles, 0.794 (95% CRI: 0.752 –0.832) for 1-year-old individuals, 0.820 (95% CRI: 0.783 –0.856) for 2-year-old individuals, and 0.868 (95% CRI: 0.852 –0.882) for ≥3-year-old individuals (adults). Mean survival was generally higher in spatial units located within the western flyway (western Germany) than in those of the eastern flyway (eastern Germany) across all age classes (Table A3-1). High probabilities (>0.95) that unit-specific survival estimates exceeded the overall mean were found only in western spatial units, while low probabilities (<0.05) occurred only in eastern units. This pattern was restricted to certain age classes and spatial units (Fig. 3 in the main text).

TABLE A3-1. Unit-specific means and temporal variability expressed as standard deviations (SD) of survival, age at first breeding, emigration, resighting probability and recovery probability, obtained under model M1. Given are posterior means and 95% credible intervals in brackets. Temporal variability is reported only for parameters for which temporal random effects were explicitly modelled, and expresses the variation of the logit of a parameter. The spatial units are ordered according to the flyways. The grey layers have no meaning but should increase the readability of the table.

Flyway	Western					Mixed			Eastern			
Spatial unit	BW	RP	BY	HE	NW	TH	NI	SH	ST	SN	MV	BB
Mean juvenile survival	0.429 (0.372-0.488)	0.454 (0.356-0.555)	0.403 (0.319-0.495)	0.498 (0.409-0.598)	0.414 (0.329-0.502)	0.438 (0.304-0.593)	0.391 (0.341-0.442)	0.362 (0.290-0.440)	0.362 (0.290-0.440)	0.307 (0.237-0.390)	0.297 (0.235-0.372)	0.254 (0.191-0.338)
Mean 1y survival	0.818 (0.778-0.854)	0.870 (0.814-0.918)	0.828 (0.744-0.896)	0.889 (0.830-0.936)	0.773 (0.658-0.872)	0.642 (0.422-0.848)	0.791 (0.700-0.870)	0.790 (0.677-0.883)	0.790 (0.677-0.883)	0.795 (0.637-0.918)	0.768 (0.628-0.869)	0.834 (0.612-0.958)
Mean 2y survival	0.852 (0.817-0.887)	0.818 (0.752-0.872)	0.845 (0.771-0.914)	0.854 (0.787-0.914)	0.913 (0.811-0.984)	0.668 (0.404-0.936)	0.786 (0.718-0.848)	0.832 (0.733-0.917)	0.832 (0.733-0.917)	0.890 (0.693-0.993)	0.767 (0.661-0.861)	0.840 (0.682-0.958)
Mean ≥3y survival	0.916 (0.898-0.933)	0.890 (0.868-0.916)	0.902 (0.864-0.937)	0.906 (0.879-0.933)	0.856 (0.806-0.901)	0.912 (0.775-0.995)	0.861 (0.817-0.903)	0.848 (0.816-0.883)	0.848 (0.816-0.883)	0.810 (0.770-0.850)	0.845 (0.820-0.872)	0.834 (0.801-0.870)

Flyway	Western					Mixed			Eastern			
Spatial unit	BW	RP	BY	HE	NW	TH	NI	SH	ST	SN	MV	BB
SD juvenile survival	0.261 (0.153-0.406)	0.604 (0.357-0.933)	0.668 (0.392-1.039)	0.567 (0.327-0.889)	0.123 (0.007-0.347)	0.335 (0.019-0.981)	0.200 (0.015-0.441)	0.439 (0.178-0.755)	0.244 (0.014-0.622)	0.455 (0.200-0.775)	0.345 (0.058-0.653)	0.518 (0.229-0.871)
SD 1y survival	0.253 (0.010-0.568)	0.258 (0.015-0.730)	0.332 (0.018-0.903)	0.357 (0.026-0.945)	0.556 (0.040-1.375)	0.475 (0.021-1.582)	0.559 (0.030-1.221)	0.586 (0.032-1.401)	0.616 (0.034-1.840)	0.267 (0.014-0.753)	0.536 (0.018-1.581)	0.568 (0.033-1.377)
SD 2y survival	0.389 (0.118-0.705)	0.348 (0.025-0.853)	0.471 (0.018-1.357)	0.579 (0.062-1.311)	1.453 (0.155-3.399)	1.128 (0.071-4.061)	0.201 (0.011-0.593)	0.622 (0.052-1.513)	2.331 (0.513-5.261)	0.365 (0.016-1.059)	1.303 (0.327-2.666)	0.556 (0.035-1.548)
SD ≥3y survival	0.358 (0.212-0.566)	0.165 (0.012-0.422)	0.559 (0.261-0.971)	0.432 (0.114-0.798)	0.487 (0.060-1.014)	1.669 (0.092-5.276)	0.546 (0.248-0.954)	0.178 (0.015-0.487)	0.299 (0.025-0.702)	0.173 (0.012-0.462)	0.228 (0.010-0.622)	0.410 (0.101-0.751)
Mean age at first breeding	2.34 (2.21-2.48)	2.48 (2.36-2.59)	2.27 (2.03-2.50)	2.11 (1.99-2.36)	2.08 (1.96-2.47)	2.20 (1.89-3.23)	2.54 (2.41-2.71)	2.65 (2.45-3.04)	3.63 (3.08-4.12)	3.40 (3.15-3.67)	3.71 (3.42-4.11)	3.61 (3.42-3.83)
Natal emigration	0.489 (0.416-0.555)	0.592 (0.502-0.666)	0.488 (0.382-0.579)	0.512 (0.412-0.596)	0.552 (0.414-0.660)	0.574 (0.391-0.730)	0.566 (0.482-0.641)	0.463 (0.333-0.568)	0.643 (0.541-0.737)	0.662 (0.569-0.749)	0.608 (0.499-0.706)	0.632 (0.524-0.722)
Breeding emigration	0.015 (0.006-0.026)	0.021 (0.006-0.045)	0.021 (0.007-0.044)	0.017 (0.003-0.034)	0.017 (0.002-0.036)	0.021 (0.003-0.064)	0.020 (0.004-0.050)	0.023 (0.007-0.058)	0.017 (0.002-0.039)	0.018 (0.003-0.038)	0.018 (0.003-0.044)	0.014 (0.002-0.028)
Mean resighting probability if seen the previous year	0.788 (0.716-0.846)	0.934 (0.870-0.973)	0.858 (0.793-0.913)	0.740 (0.656-0.822)	0.575 (0.345-0.813)	0.306 (0.009-0.899)	0.590 (0.536-0.644)	0.657 (0.576-0.742)	0.721 (0.664-0.779)	0.640 (0.558-0.716)	0.619 (0.558-0.672)	0.675 (0.615-0.733)

Flyway	Western					Mixed			Eastern			
Spatial unit	BW	RP	BY	HE	NW	TH	NI	SH	ST	SN	MV	BB
Mean resighting probability if not seen the previous year	0.296 (0.196-0.404)	0.407 (0.289-0.513)	0.336 (0.216-0.468)	0.235 (0.164-0.317)	0.161 (0.103-0.228)	0.183 (0.079-0.326)	0.279 (0.224-0.338)	0.335 (0.278-0.396)	0.430 (0.341-0.521)	0.361 (0.298-0.425)	0.382 (0.312-0.453)	0.277 (0.233-0.326)
SD resighting probability if seen the previous year	0.834 (0.590-1.194)	1.538 (0.870-2.654)	0.840 (0.509-1.363)	0.938 (0.631-1.419)	2.113 (1.241-3.585)	5.505 (1.087-9.709)	0.303 (0.048-0.658)	0.634 (0.321-1.059)	0.292 (0.020-0.680)	0.739 (0.486-1.101)	0.232 (0.015-0.616)	0.538 (0.316-0.828)
SD resighting probability if not seen the previous year	1.160 (0.831-1.631)	0.827 (0.311-1.443)	1.135 (0.718-1.809)	0.906 (0.629-1.316)	0.798 (0.423-1.338)	0.939 (0.346-1.820)	0.504 (0.313-0.775)	0.241 (0.033-0.512)	0.332 (0.025-0.846)	0.426 (0.230-0.677)	0.329 (0.061-0.634)	0.318 (0.138-0.542)
1y recovery probability	0.096 (0.086-0.107)	0.116 (0.098-0.137)	0.118 (0.104-0.134)	0.117 (0.100-0.137)	0.080 (0.067-0.095)	0.072 (0.056-0.092)	0.059 (0.054-0.066)	0.079 (0.069-0.090)	0.055 (0.047-0.063)	0.048 (0.043-0.054)	0.046 (0.041-0.053)	0.036 (0.031-0.043)
2y recovery probability	0.097 (0.090-0.106)	0.112 (0.095-0.134)	0.117 (0.089-0.149)	0.119 (0.096-0.149)	0.082 (0.067-0.102)	0.072 (0.042-0.115)	0.058 (0.045-0.069)	0.081 (0.064-0.105)	0.048 (0.029-0.063)	0.040 (0.025-0.054)	0.036 (0.012-0.079)	0.020 (0.008-0.035)
3y recovery probability	0.097 (0.080-0.113)	0.097 (0.068-0.122)	0.112 (0.076-0.147)	0.100 (0.075-0.126)	0.079 (0.054-0.101)	0.073 (0.050-0.101)	0.060 (0.052-0.068)	0.078 (0.057-0.100)	0.043 (0.022-0.062)	0.051 (0.041-0.062)	0.044 (0.032-0.057)	0.024 (0.011-0.037)
≥4y recovery probability	0.101 (0.084-0.126)	0.102 (0.084-0.132)	0.118 (0.097-0.143)	0.106 (0.091-0.125)	0.088 (0.069-0.120)	0.071 (0.040-0.114)	0.062 (0.053-0.074)	0.080 (0.067-0.095)	0.061 (0.050-0.075)	0.056 (0.046-0.066)	0.048 (0.040-0.058)	0.040 (0.032-0.050)

Flyway	Western					Mixed			Eastern			
Spatial unit	BW	RP	BY	HE	NW	TH	NI	SH	ST	SN	MV	BB
Recovery probability in Germany	0.107 (0.053-0.178)	0.085 (0.034-0.161)	0.106 (0.052-0.170)	0.085 (0.046-0.148)	0.098 (0.048-0.180)	0.073 (0.043-0.109)	0.067 (0.041-0.096)	0.061 (0.020-0.110)	0.072 (0.047-0.098)	0.064 (0.043-0.079)	0.053 (0.027-0.069)	0.056 (0.033-0.073)
Recovery probability outside Germany	0.087 (0.013-0.150)	0.144 (0.047-0.221)	0.144 (0.005-0.306)	0.149 (0.062-0.220)	0.069 (0.010-0.117)	0.072 (0.006-0.172)	0.050 (0.010-0.088)	0.114 (0.019-0.200)	0.029 (0.002-0.069)	0.020 (0.001-0.062)	0.030 (0.001-0.099)	0.009 (0.000-0.034)

1.2 Age at first breeding

The overall arithmetic mean age at first breeding across all spatial units was 2.75 years (95% CRI: 2.67–2.86). Mean values at the spatial-unit level were higher in the eastern flyway than in the western or mixed flyways (Table A3-1). In mixed flyway units, estimates were closer to those of the western than the eastern flyway (Table A3-1). The probability that a unit-specific mean age at first breeding exceeded the global mean was very high in the eastern and very low in the western flyway (Fig. 3 in the main text).

1.3 Emigration

Across all spatial units, the mean probability of natal emigration was estimated at 0.566 (95% CRI: 0.496–0.634). Individuals from the eastern flyway were more likely to emigrate from their natal unit than those from the western flyway (Table A3-1). Breeding emigration remained consistently low throughout the study area, with an overall mean of 0.017 (95% CRI: 0.006–0.030; Table A3-1).

1.4 Resighting probability

Resighting probability was higher for individuals observed in the previous year than for those not seen the year before (Table A3-1). Mean resighting probabilities differed among spatial units, yet no consistent spatial pattern was apparent (Table A3-1).

2.5 Dead-recovery probability

The recovery probabilities inside and outside of Germany were both lower in spatial units associated with the eastern flyway than in those of the western flyway (Table A3-1). In the east, recovery probabilities were lowest for 1- and 2-year-old individuals and highest for adults, whereas in western Germany no consistent age-related pattern was apparent.

2. Results from model M2 (standard ICAR)

2.1 Survival

The overall mean annual survival probability across spatial units and years was 0.368 (95% CRI: 0.845–0.890) for juveniles, 0.812 (95% CRI: 0.781–0.839) for 1-year-old individuals, 0.829 (95% CRI: 0.806–0.852) for 2-year individuals, and 0.870 (95% CRI: 0.859–0.882) for ≥3-year-old individuals (adults). Mean survival was higher in spatial units located within the western flyway (western Germany) than in those of the eastern flyway (eastern Germany) across all age classes (Table A3-2). In both juveniles and adults, the probability of unit-specific survival estimates exceeding the overall mean was high in the west and low in the east. However, this west–east contrast was slightly weaker than that observed under M3 (in particular for juveniles; Fig. 3 in the main text). In contrast, survival of 1y and 2y individuals showed only limited spatial variation (Table A3-2).

TABLE A3-2. Unit-specific means and temporal variability expressed as standard deviations (SD) of survival, age at first breeding, emigration, resighting probability and recovery probability, obtained under model M2. Given are posterior means and 95% credible intervals in brackets. Temporal variability is reported only for parameters for which temporal random effects were explicitly modelled, and expresses the variation of the logit of a parameter. The spatial units are ordered according to the flyways. The grey layers have no meaning but should increase the readability of the table.

Flyway	Western					Mixed			Eastern			
Spatial unit	BW	RP	BY	HE	NW	TH	NI	SH	ST	SN	MV	BB
Mean juvenile survival	0.407 (0.364-0.457)	0.410 (0.351-0.482)	0.387 (0.334-0.446)	0.415 (0.364-0.481)	0.394 (0.333-0.464)	0.381 (0.318-0.459)	0.375 (0.334-0.420)	0.350 (0.290-0.411)	0.340 (0.277-0.397)	0.331 (0.270-0.387)	0.304 (0.237-0.370)	0.334 (0.275-0.388)
Mean 1y survival	0.815 (0.785-0.844)	0.825 (0.789-0.871)	0.814 (0.771-0.851)	0.821 (0.787-0.862)	0.812 (0.761-0.851)	0.806 (0.742-0.845)	0.808 (0.761-0.842)	0.804 (0.739-0.849)	0.808 (0.747-0.848)	0.806 (0.744-0.845)	0.806 (0.732-0.851)	0.805 (0.732-0.845)
Mean 2y survival	0.839 (0.812-0.869)	0.832 (0.799-0.863)	0.832 (0.799-0.865)	0.834 (0.806-0.864)	0.835 (0.799-0.879)	0.826 (0.775-0.859)	0.823 (0.780-0.852)	0.825 (0.770-0.867)	0.828 (0.784-0.864)	0.822 (0.769-0.855)	0.825 (0.770-0.862)	0.824 (0.770-0.857)
Mean ≥3y survival	0.913 (0.896-0.929)	0.892 (0.870-0.915)	0.892 (0.865-0.918)	0.895 (0.873-0.915)	0.870 (0.833-0.902)	0.871 (0.830-0.908)	0.862 (0.834-0.889)	0.850 (0.819-0.881)	0.835 (0.797-0.869)	0.851 (0.825-0.875)	0.844 (0.811-0.877)	0.839 (0.810-0.868)

Flyway	Western					Mixed			Eastern			
Spatial unit	BW	RP	BY	HE	NW	TH	NI	SH	ST	SN	MV	BB
SD juvenile survival	0.245 (0.143-0.384)	0.578 (0.337-0.878)	0.646 (0.387-1.002)	0.497 (0.272-0.799)	0.180 (0.065-0.377)	0.383 (0.087-0.971)	0.231 (0.079-0.457)	0.430 (0.185-0.736)	0.294 (0.085-0.612)	0.480 (0.236-0.779)	0.369 (0.130-0.677)	0.516 (0.273-0.845)
SD 1y survival	0.282 (0.078-0.564)	0.325 (0.073-0.786)	0.355 (0.074-0.857)	0.347 (0.073-0.855)	0.644 (0.111-1.441)	0.758 (0.109-2.175)	0.600 (0.144-1.214)	0.597 (0.095-1.345)	0.610 (0.094-1.643)	0.326 (0.077-0.806)	0.540 (0.089-1.343)	0.604 (0.095-1.426)
SD 2y survival	0.387 (0.145-0.716)	0.368 (0.087-0.827)	0.469 (0.099-1.140)	0.579 (0.129-1.243)	1.091 (0.212-2.496)	1.621 (0.210-4.230)	0.300 (0.073-0.699)	0.633 (0.124-1.435)	1.823 (0.494-3.759)	0.472 (0.102-1.137)	1.193 (0.367-2.254)	0.608 (0.107-1.447)
SD ≥3y survival	0.357 (0.212-0.567)	0.218 (0.080-0.459)	0.534 (0.262-0.938)	0.401 (0.147-0.715)	0.523 (0.140-1.049)	0.994 (0.138-3.165)	0.524 (0.240-0.922)	0.243 (0.078-0.534)	0.382 (0.103-0.780)	0.217 (0.074-0.462)	0.318 (0.094-0.681)	0.428 (0.160-0.759)
Mean age at first breeding	2.32 (2.19-2.45)	2.46 (2.31-2.59)	2.28 (2.07-2.49)	2.12 (2.00-2.30)	2.10 (1.99-2.37)	2.25 (2.00-2.85)	2.50 (2.34-2.65)	2.61 (2.42-2.85)	3.43 (2.97-3.87)	3.29 (2.97-3.60)	3.56 (3.24-3.84)	3.55 (3.26-3.79)
Natal emigration	0.459 (0.399-0.523)	0.541 (0.458-0.616)	0.457 (0.365-0.538)	0.404 (0.306-0.495)	0.535 (0.417-0.637)	0.617 (0.434-0.759)	0.572 (0.501-0.635)	0.445 (0.326-0.545)	0.678 (0.590-0.751)	0.720 (0.654-0.775)	0.652 (0.558-0.729)	0.675 (0.604-0.735)
Breeding emigration	0.015 (0.006-0.025)	0.022 (0.007-0.046)	0.019 (0.006-0.037)	0.014 (0.003-0.027)	0.017 (0.003-0.039)	0.020 (0.004-0.057)	0.020 (0.005-0.048)	0.022 (0.007-0.049)	0.019 (0.004-0.046)	0.019 (0.005-0.042)	0.019 (0.004-0.045)	0.014 (0.002-0.029)
Mean resighting probability if seen the previous year	0.788 (0.716-0.846)	0.933 (0.868-0.972)	0.858 (0.792-0.912)	0.742 (0.654-0.824)	0.573 (0.336-0.812)	0.284 (0.009-0.874)	0.587 (0.530-0.641)	0.656 (0.578-0.743)	0.718 (0.662-0.775)	0.638 (0.557-0.715)	0.615 (0.558-0.668)	0.673 (0.613-0.734)

Flyway	Western					Mixed			Eastern			
Spatial unit	BW	RP	BY	HE	NW	TH	NI	SH	ST	SN	MV	BB
Mean resighting probability if not seen the previous year	0.290 (0.195-0.401)	0.401 (0.286-0.504)	0.334 (0.212-0.471)	0.234 (0.162-0.313)	0.159 (0.099-0.224)	0.181 (0.083-0.313)	0.275 (0.221-0.333)	0.325 (0.271-0.383)	0.413 (0.333-0.495)	0.351 (0.289-0.415)	0.365 (0.297-0.433)	0.271 (0.227-0.318)
SD resighting probability if seen the previous year	0.837 (0.589-1.198)	1.520 (0.866-2.596)	0.833 (0.508-1.335)	0.945 (0.611-1.437)	2.117 (1.264-3.608)	5.589 (1.162-9.755)	0.306 (0.042-0.646)	0.639 (0.313-1.084)	0.296 (0.024-0.668)	0.734 (0.472-1.099)	0.222 (0.017-0.587)	0.537 (0.312-0.849)
SD resighting probability if not seen the previous year	1.173 (0.835-1.649)	0.843 (0.405-1.472)	1.157 (0.733-1.818)	0.901 (0.626-1.308)	0.819 (0.451-1.357)	0.858 (0.260-1.708)	0.490 (0.303-0.759)	0.239 (0.023-0.512)	0.295 (0.024-0.739)	0.424 (0.226-0.688)	0.323 (0.063-0.620)	0.323 (0.140-0.543)
1y recovery probability	0.092 (0.084-0.101)	0.106 (0.093-0.122)	0.115 (0.103-0.129)	0.103 (0.091-0.117)	0.077 (0.065-0.090)	0.074 (0.057-0.093)	0.060 (0.054-0.066)	0.078 (0.069-0.088)	0.056 (0.049-0.064)	0.049 (0.044-0.055)	0.047 (0.042-0.054)	0.035 (0.031-0.039)
2y recovery probability	0.098 (0.090-0.108)	0.105 (0.085-0.120)	0.110 (0.086-0.131)	0.100 (0.077-0.118)	0.087 (0.068-0.113)	0.078 (0.049-0.127)	0.058 (0.047-0.068)	0.081 (0.066-0.103)	0.047 (0.030-0.062)	0.044 (0.031-0.056)	0.027 (0.013-0.047)	0.024 (0.014-0.034)
3y recovery probability	0.101 (0.088-0.114)	0.111 (0.087-0.133)	0.105 (0.072-0.132)	0.111 (0.088-0.138)	0.071 (0.041-0.096)	0.076 (0.052-0.106)	0.061 (0.053-0.070)	0.077 (0.058-0.097)	0.039 (0.020-0.059)	0.051 (0.043-0.061)	0.042 (0.029-0.052)	0.026 (0.016-0.036)
≥4y recovery probability	0.109 (0.089-0.128)	0.118 (0.092-0.151)	0.121 (0.101-0.144)	0.112 (0.094-0.135)	0.101 (0.077-0.132)	0.071 (0.036-0.110)	0.063 (0.053-0.074)	0.080 (0.068-0.095)	0.063 (0.052-0.076)	0.054 (0.045-0.064)	0.050 (0.042-0.058)	0.037 (0.032-0.044)

Flyway	Western					Mixed			Eastern			
Spatial unit	BW	RP	BY	HE	NW	TH	NI	SH	ST	SN	MV	BB
Recovery probability in Germany	0.133 (0.073-0.184)	0.128 (0.063-0.195)	0.129 (0.067-0.173)	0.125 (0.070-0.181)	0.134 (0.068-0.197)	0.076 (0.042-0.112)	0.070 (0.042-0.098)	0.065 (0.023-0.112)	0.078 (0.052-0.100)	0.061 (0.041-0.077)	0.058 (0.044-0.070)	0.050 (0.034-0.065)
Recovery probability outside Germany	0.058 (0.006-0.123)	0.085 (0.009-0.168)	0.082 (0.004-0.234)	0.081 (0.015-0.154)	0.043 (0.004-0.095)	0.074 (0.005-0.185)	0.048 (0.010-0.087)	0.105 (0.014-0.192)	0.022 (0.001-0.060)	0.028 (0.002-0.069)	0.016 (0.001-0.047)	0.014 (0.001-0.032)

2.2 Age at first breeding

The overall mean age at first breeding across all spatial units was 2.63 years (95% CRI: 2.48–2.74). Mean values at the spatial-unit level were higher in the eastern flyway than in the western or mixed flyways (Table A3-2). In mixed flyway units, estimates were closer to those of the western than the eastern flyway (Table A3-2). The probability that a unit-specific mean age at first breeding exceeded the global mean was very high in the eastern and very low in the western flyway (Fig. 3 in the main text).

2.3 Emigration

Across all spatial units, the mean probability of natal emigration was estimated at 0.565 (95% CRI: 0.479–0.648). Individuals from the eastern flyway were more likely to emigrate from their natal unit than those from the western flyway (Table A3-2). Breeding emigration remained consistently low throughout the study area, with an overall mean of 0.017 (95% CRI: 0.007–0.029; Table A3-2).

2.4 Resighting probability

Resighting probability was higher for individuals observed in the previous year than for those not seen the year before (Table A3-2). Mean resighting probabilities differed among spatial units, but no consistent spatial pattern was apparent (Table A3-2).

2.5 Dead-recovery probability

The recovery probabilities inside and outside of Germany were both lower in spatial units associated with the eastern flyway than in those of the western flyway (Table A3-2). In the east, recovery probabilities were lowest for 1- and 2-year-old individuals and highest for adults, whereas in western Germany no consistent age-related pattern was apparent (Table A3-2).
