# Supplementary material for: Assessment of large‐scale spatial variation in age‐specific survival and age at first breeding in a long‐lived species
Source: J Anim Ecol. 2026 Jun 5;95(7):1260–72. doi: 10.1111/1365-2656.70291 (PMC13322180; doi:10.1111/1365-2656.70291)
Supplement: Supplementary file 4 — Appendix S4. Simulation study to assess the estimation of dead‐recovery probabilities. [file JANE-95-1260-s002.pdf]

APPENDIX S4 – SIMULATION STUDY TO ASSESS THE ESTIMATION OF DEAD RECOVERY PROBABILITIES

To assess the ability of our modelling approach to correctly estimate age-specific dead recovery probabilities, we conducted a simulation study. The simulated population included four age classes for survival and dead recovery, as well as delayed recruitment into the breeding population. In contrast to the model in the paper and for simplicity, we assumed constant probabilities of survival, recruitment, and resighting over time. We also have not considered any spatial variation. As in our model, age-dependent recovery probabilities were considered to be dependent on the two age-independent recovery probabilities rb and rnb, that represent respectively the probability to be found dead inside and outside of the breeding area, and on the age-dependent proportion of birds recovered outside the breeding area $prop_a$. Thus, age-dependent recovery probabilities to be used for data simulation were not independently generated, but calculated from rb, rnb and $prop_a$.

The parameters used in the simulations were:

- Survival probabilities: juvenile = 0.3, 1y = 0.7, 2y = 0.8, ≥3y = 0.9
- Recruitment probabilities: 1y = 0.05, 2y = 0.25, 3y = 0.5, 4y = 0.8, 5y = 1.0
- Emigration probabilities: natal = 0.5; breeding = 0.02
- Resighting probability: 0.5
- Dead recovery probabilities: inside the breeding area = 0.2; outside the breeding area = 0.05
- Proportion of birds recovered outside the breeding area: 1y = 0.4, 2y = 0.7, 3y = 0.5, ≥4y = 0.1

These values yielded overall dead recovery probabilities of: 1y = 0.14; 2y = 0.095; 3y; = 0.125; ≥4y = 0.185. We simulated a 20-year study period during which 200 juveniles were marked annually. In addition, 20 breeding 2y and 20 breeding ≥3y were marked in the first study year. The sample sizes, study duration, and parameter values were chosen to match approximately the characteristics of a typical spatial unit from our empirical dataset. 50 simulation runs were performed.

The simulations produced individual encounter histories, which were then formatted into m-arrays. We preformed Bayesian inference using NIMBLE (version 1.3; Valpine et al., 2017). For each run, the model was executed with 10,000 iterations across four chains, including a burn-in of 1,000 and a thinning interval of 10. Convergence was verified for all simulation replicates. This simulation framework allowed us to evaluate the model's ability to recover the dead recovery probabilities across the four defined age classes under realistic demographic assumptions. We also analysed the 50 simulated data sets with a model ignoring age dependence in dead recovery probability (i.e., treating dead recovery probability to be the same for all four age classes), to assess the bias generated when age-dependence in recovery is ignored.

In both scenarios, we calculated the mean absolute bias (the average absolute difference between estimated and true values, regardless of the direction of the error) and the coverage (the proportion of simulation runs whose estimated 95% credible interval included the true value) of survival and the dead recovery probabilities across all simulation runs. Results are shown in Table A4-1.

TABLE A4-1. For each parameter and each model (with or without age dependence in recovery probability), mean absolute bias (bias) and coverage is given across 50 simulation runs.

	Including age dependence		Without age dependence	
Parameter	Bias	Coverage	Bias	Coverage
Juvenile survival	0.029	0.92	0.023	0.96
1y survival	0.047	0.98	0.085	0.46
2y survival	0.031	0.96	0.038	0.94
≥3y survival	0.012	0.94	0.012	0.94
1y recovery	0.007	0.96	0.006	1.00
2y recovery	0.022	0.98	0.047	0.00

3y recovery	0.014	1.00	0.017	0.00
≥4y recovery	0.021	0.98	0.044	0.00

Our results show that the model accounting for age-dependent dead recovery probabilities (structured through age-specific probabilities of being recovered inside or outside Germany) provide accurate estimates (low relative bias and low absolute error) and appropriate coverage (close to 0.95) for both survival and dead recovery parameters. In contrast, ignoring age dependence in dead recovery led to larger bias in recovery probabilities and to zero coverage for age classes ≥ 2 years, the latter partly resulting from an underestimation of uncertainty. This pattern propagated to survival estimates, notably for 1-year-olds, where coverage dropped to 0.46 and bias increased markedly.

These simulation results demonstrate that our modelling approach provides reliable estimates of age-specific survival and recovery probabilities when it is not possible to estimate separate dead recovery probabilities for each age class. Compared to a model that entirely ignores age dependence in dead recovery, our approach leads to more accurate survival estimates, especially for early age classes.
