# Supplementary material for: Assessment of large‐scale spatial variation in age‐specific survival and age at first breeding in a long‐lived species
Source: J Anim Ecol. 2026 Jun 5;95(7):1260–72. doi: 10.1111/1365-2656.70291 (PMC13322180; doi:10.1111/1365-2656.70291)
Supplement: Supplementary file 5 — Figure S1. Box plots of parameter uncertainty expressed as the width of the 95% credible interval (CRI) under each model (M1, M2, M3). Figure S2. Posterior means (bold lines) and 95% credible intervals (bands) of annual survival probability (4 age classes) across time (2000–2022) for each spatial unit (see Table 1 for unit codes) obtained from model M3. The figure is structured by flyway. Figure S3. Posterior means (bold lines) and 95% credible interval (bands) of time‐dependent age at first breeding (2001–2023) for each spatial unit obtained from model M3. Asterisks in MV indicate estimates with R‐hat >1.1. Table S1. Unit‐specific means and temporal variability (standard deviations, SD) of survival, age at first breeding, resighting and recovery probabilities obtained from model M3. Given are posterior means and 95% CRI in brackets. Table S2. Posterior probability per spatial unit that the temporal range in survival (2000–2022) for one age class exceeds that of another (obtained from model M3). [file JANE-95-1260-s004.pdf]

SUPPLEMENTARY FIGURES AND TABLES

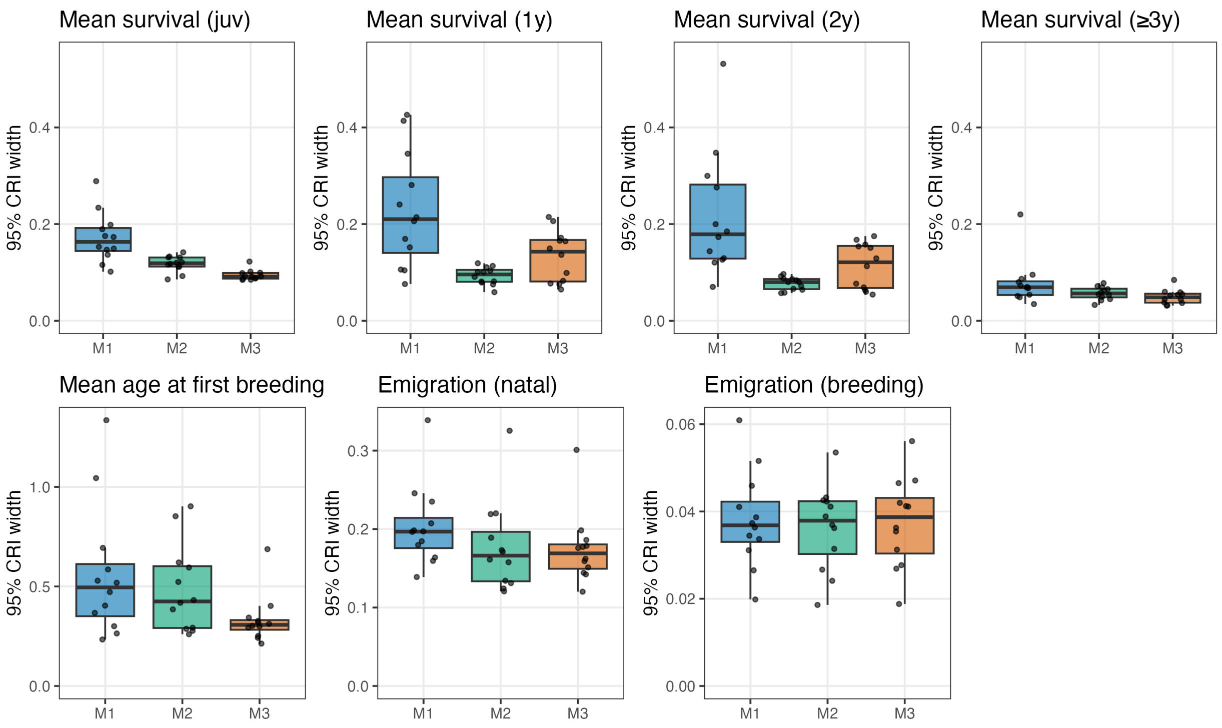

FIGURE S1. Box plots of parameter uncertainty expressed as the width of the 95% credible interval (CRI) under each model (M1, M2, M3). Boxes represent the median and interquartile range of spatial units; whiskers extend to 1.5 times the interquartile range, and individual spatial units are shown as points. Panels correspond to seven parameters: mean survival (juvenile, 1y, 2y, ≥3y), mean age at first breeding, and natal and breeding emigration.

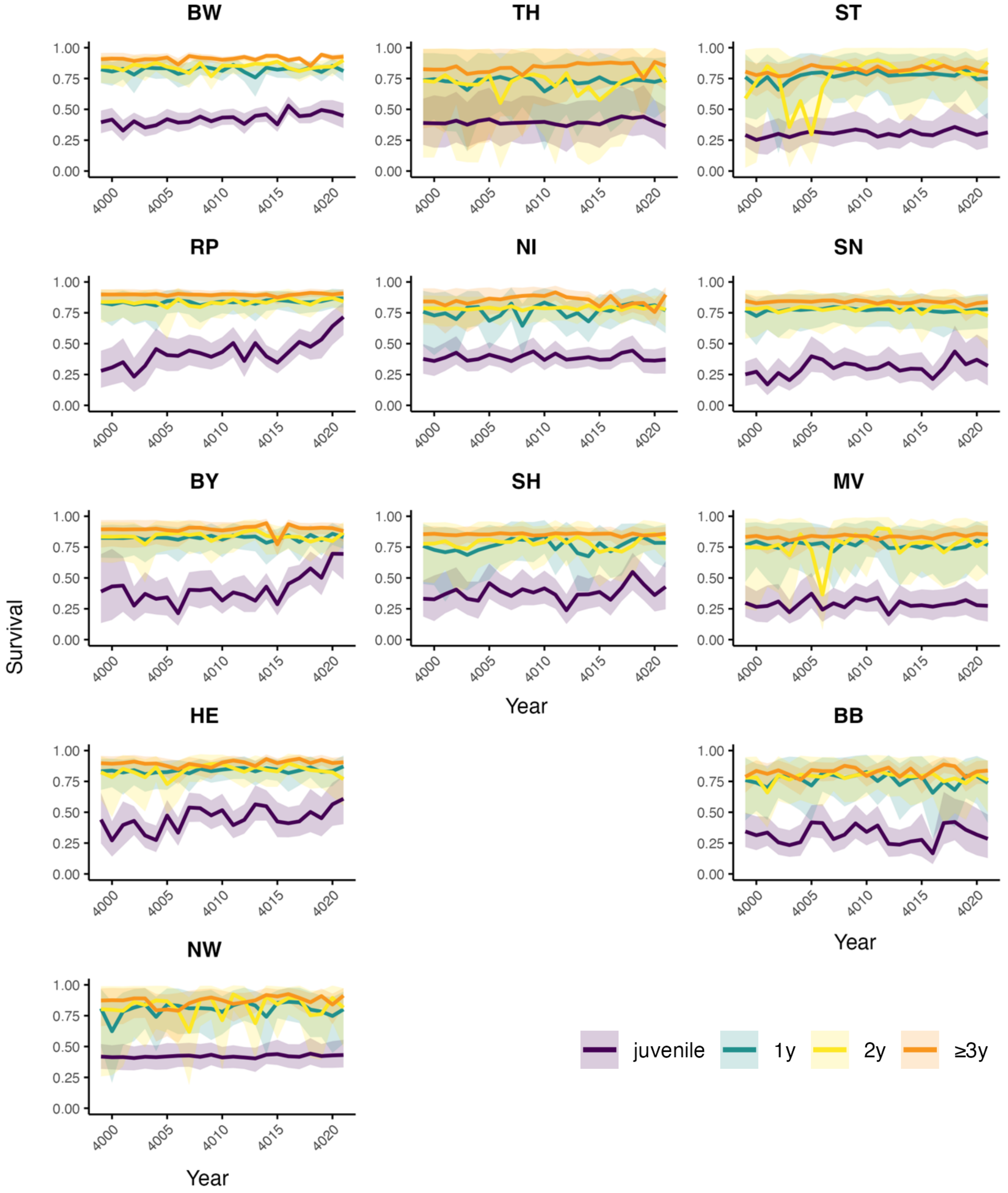

FIGURE S2. Posterior means (bold lines) and 95% credible intervals (bands) of annual survival probability (4 age classes) across time (2000-2022) for each spatial unit (see Table 1 for unit codes) obtained from model M3. The figure is structured by flyway.

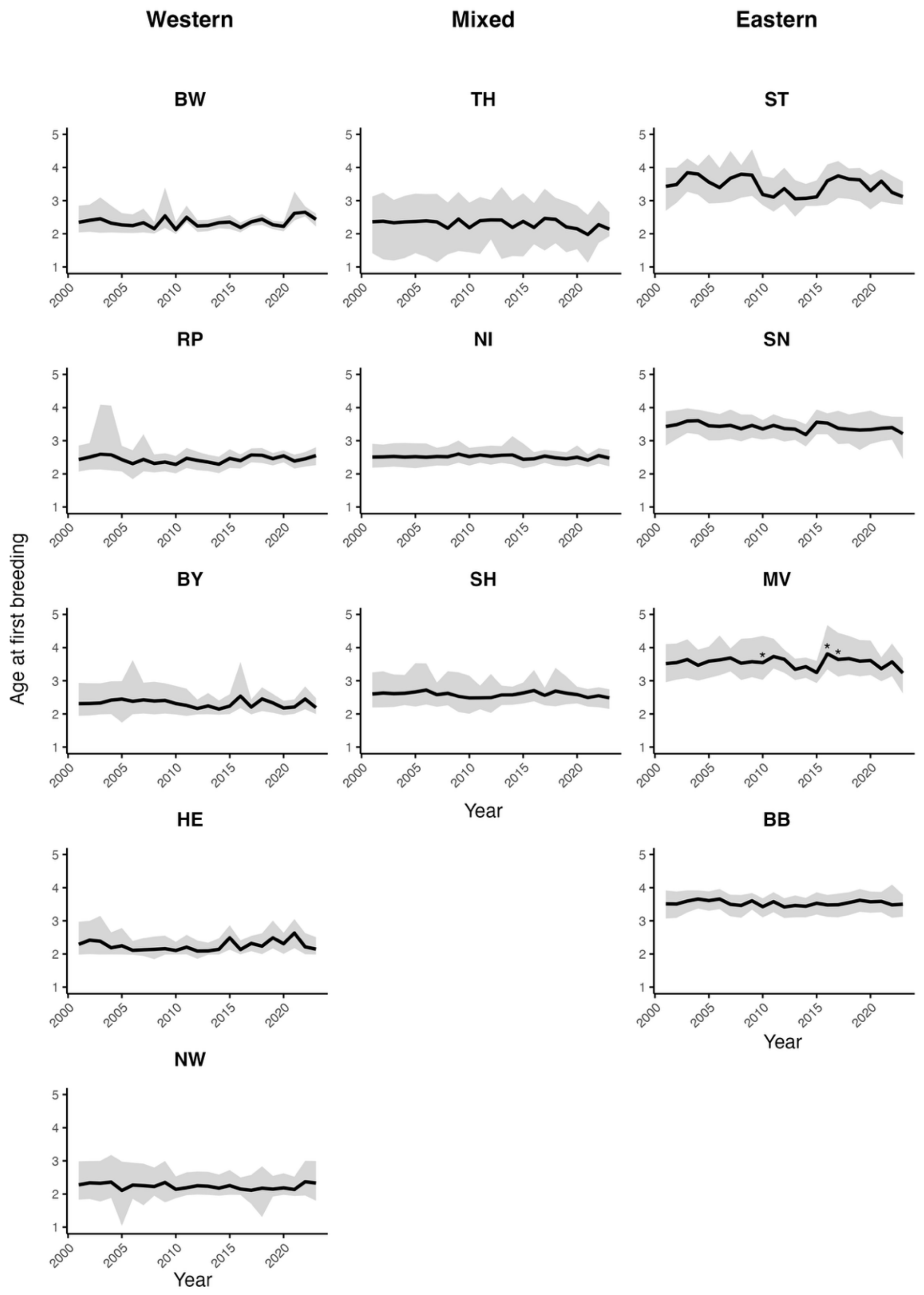

FIGURE S3. Posterior means (bold lines) and 95% credible interval (bands) of time-dependent age at first breeding (2001-2023) for each spatial unit obtained from model M3. Asterisks in MV indicate estimates with R-hat > 1.1.

TABLE S1. Unit-specific means and temporal variability (standard deviations, SD) of survival, age at first breeding, resighting and recovery probabilities obtained from model M3. Given are posterior means and 95% CRI in brackets. Temporal variability is reported only for parameters for which temporal random effects were explicitly modelled, and expresses the variation of the logit of a parameter. The flyway associated with each unit is indicated as W (Western), M (Mixed), or E (Eastern). See Table 1 for the unit codes. The grey layers have no meaning but should increase the readability of the table.

Flyway	W					M			E			
Spatial unit	BW	RP	BY	HE	NW	TH	NI	SH	ST	SN	MV	BB
Mean juvenile survival	0.422 (0.382-0.467)	0.424 (0.380-0.472)	0.421 (0.376-0.467)	0.426 (0.385-0.472)	0.421 (0.370-0.472)	0.390 (0.334-0.456)	0.386 (0.343-0.431)	0.381 (0.332-0.430)	0.300 (0.258-0.349)	0.298 (0.258-0.343)	0.289 (0.240-0.340)	0.298 (0.258-0.345)
Mean 1y survival	0.828 (0.793-0.858)	0.839 (0.803-0.880)	0.832 (0.790-0.872)	0.839 (0.804-0.880)	0.830 (0.773-0.872)	0.752 (0.621-0.836)	0.770 (0.696-0.833)	0.775 (0.698-0.848)	0.781 (0.689-0.855)	0.778 (0.686-0.850)	0.781 (0.669-0.875)	0.779 (0.684-0.856)
Mean 2y survival	0.849 (0.822-0.876)	0.847 (0.812-0.876)	0.848 (0.812-0.881)	0.849 (0.819-0.879)	0.852 (0.816-0.892)	0.782 (0.678-0.853)	0.791 (0.731-0.844)	0.797 (0.731-0.860)	0.799 (0.714-0.872)	0.794 (0.712-0.866)	0.799 (0.709-0.876)	0.798 (0.717-0.868)
Mean ≥3y survival	0.908 (0.893-0.924)	0.900 (0.880-0.917)	0.905 (0.886-0.923)	0.903 (0.886-0.918)	0.896 (0.862-0.916)	0.862 (0.822-0.906)	0.860 (0.830-0.890)	0.857 (0.827-0.886)	0.830 (0.799-0.854)	0.838 (0.817-0.862)	0.837 (0.811-0.863)	0.834 (0.812-0.856)

Flyway	W					M			E			
Spatial unit	BW	RP	BY	HE	NW	TH	NI	SH	ST	SN	MV	BB
SD juvenile survival	0.250 (0.143-0.393)	0.586 (0.344-0.906)	0.662 (0.408-1.014)	0.519 (0.283-0.814)	0.169 (0.048-0.399)	0.350 (0.067-0.923)	0.216 (0.061-0.443)	0.423 (0.169-0.710)	0.269 (0.064-0.614)	0.441 (0.193-0.754)	0.348 (0.103-0.643)	0.503 (0.261-0.815)
SD 1y survival	0.297 (0.087-0.588)	0.324 (0.078-0.779)	0.374 (0.082-0.886)	0.365 (0.081-0.880)	0.689 (0.131-1.507)	0.656 (0.108-1.885)	0.545 (0.130-1.106)	0.600 (0.119-1.318)	0.574 (0.106-1.540)	0.327 (0.080-0.784)	0.539 (0.102-1.335)	0.598 (0.110-1.410)
SD 2y survival	0.384 (0.135-0.713)	0.390 (0.097-0.888)	0.507 (0.107-1.201)	0.584 (0.141-1.215)	1.132 (0.232-2.496)	1.352 (0.175-3.956)	0.273 (0.072-0.636)	0.616 (0.126-1.406)	1.746 (0.419-3.709)	0.455 (0.098-1.089)	1.127 (0.308-2.244)	0.583 (0.115-1.369)
SD ≥3y survival	0.360 (0.209-0.581)	0.217 (0.062-0.465)	0.538 (0.257-0.941)	0.399 (0.133-0.729)	0.632 (0.158-1.169)	0.973 (0.118-3.017)	0.528 (0.235-0.939)	0.227 (0.062-0.521)	0.355 (0.084-0.765)	0.213 (0.059-0.484)	0.273 (0.068-0.637)	0.397 (0.126-0.711)
Mean age at first breeding	2.31 (2.20-2.42)	2.41 (2.26-2.55)	2.27 (2.10-2.43)	2.22 (2.06-2.37)	2.22 (2.02 -2.41)	2.34 (2.01-2.70)	2.50 (2.37-2.62)	2.57 (2.42-2.72)	3.50 (3.32-3.67)	3.47 (3.30-3.60)	3.55 (3.40-3.72)	3.53 (3.41-3.65)
Natal emigration	0.485 (0.425-0.545)	0.562 (0.489-0.632)	0.495 (0.411-0.573)	0.441 (0.348-0.526)	0.570 (0.468-0.654)	0.591 (0.427-0.728)	0.555 (0.478-0.622)	0.463 (0.356-0.555)	0.625 (0.533-0.709)	0.672 (0.592-0.743)	0.616 (0.520-0.698)	0.633 (0.549-0.708)
Breeding emigration	0.014 (0.006-0.024)	0.026 (0.009-0.050)	0.022 (0.007-0.044)	0.016 (0.003-0.031)	0.021 (0.004-0.051)	0.020 (0.003-0.059)	0.020 (0.004-0.046)	0.023 (0.007-0.053)	0.018 (0.003-0.044)	0.016 (0.003-0.034)	0.017 (0.003-0.038)	0.014 (0.002-0.029)
Mean resighting probability if seen the previous year	0.787 (0.717-0.843)	0.934 (0.872-0.973)	0.858 (0.795-0.914)	0.741 (0.650-0.821)	0.572 (0.346-0.802)	0.289 (0.008-0.892)	0.589 (0.532-0.641)	0.658 (0.575-0.746)	0.719 (0.661-0.775)	0.641 (0.558-0.718)	0.615 (0.556-0.668)	0.673 (0.613-0.733)

Flyway	W					M			E			
Spatial unit	BW	RP	BY	HE	NW	TH	NI	SH	ST	SN	MV	BB
Mean resighting probability if not seen the previous year	0.288 (0.191-0.397)	0.400 (0.280-0.505)	0.336 (0.216-0.466)	0.239 (0.168-0.319)	0.164 (0.102-0.236)	0.184 (0.086-0.325)	0.274 (0.220-0.334)	0.318 (0.265-0.373)	0.413 (0.335-0.493)	0.364 (0.303-0.426)	0.357 (0.292-0.425)	0.270 (0.227-0.318)
SD resighting probability if seen the previous year	0.836 (0.586-1.224)	1.516 (0.857-2.552)	0.840 (0.508-1.368)	0.941 (0.618-1.433)	2.109 (1.283-3.475)	5.720 (1.179-9.756)	0.308 (0.038-0.648)	0.647 (0.322-1.114)	0.298 (0.023-0.674)	0.738 (0.479-1.100)	0.225 (0.012-0.622)	0.539 (0.314-0.837)
SD resighting probability if not seen the previous year	1.169 (0.831-1.645)	0.817 (0.312-1.484)	1.135 (0.715-1.748)	0.893 (0.617-1.298)	0.853 (0.484-1.384)	0.884 (0.289-1.753)	0.494 (0.312-0.755)	0.233 (0.026-0.507)	0.306 (0.022-0.770)	0.410 (0.215-0.663)	0.361 (0.076-0.668)	0.321 (0.145-0.536)
1y recovery probability	0.094 (0.086-0.104)	0.109 (0.095-0.125)	0.118 (0.105-0.132)	0.106 (0.093-0.120)	0.080 (0.068-0.093)	0.073 (0.057-0.092)	0.059 (0.053-0.065)	0.079 (0.070-0.090)	0.055 (0.048-0.062)	0.048 (0.043-0.054)	0.047 (0.041-0.053)	0.034 (0.030-0.039)
2y recovery probability	0.098 (0.091-0.107)	0.107 (0.089-0.122)	0.115 (0.091-0.139)	0.104 (0.082-0.124)	0.088 (0.071-0.113)	0.074 (0.047-0.112)	0.056 (0.043-0.066)	0.079 (0.062-0.096)	0.046 (0.030-0.061)	0.041 (0.028-0.052)	0.025 (0.012-0.046)	0.023 (0.014-0.033)
3y recovery probability	0.100 (0.085-0.113)	0.111 (0.088-0.136)	0.110 (0.076-0.139)	0.110 (0.086-0.136)	0.073 (0.042-0.096)	0.076 (0.053-0.103)	0.060 (0.051-0.070)	0.076 (0.057-0.094)	0.038 (0.020-0.057)	0.051 (0.042-0.061)	0.041 (0.029-0.053)	0.025 (0.015-0.036)
≥4y recovery probability	0.104 (0.087-0.126)	0.116 (0.092-0.148)	0.121 (0.101-0.145)	0.110 (0.094-0.133)	0.100 (0.077-0.131)	0.070 (0.036-0.105)	0.064 (0.055-0.076)	0.080 (0.069-0.095)	0.063 (0.052-0.077)	0.056 (0.047-0.066)	0.051 (0.043-0.059)	0.038 (0.032-0.046)

Flyway	W					M			E			
Spatial unit	BW	RP	BY	HE	NW	TH	NI	SH	ST	SN	MV	BB
Recovery probability in Germany	0.119 (0.062-0.178)	0.122 (0.062-0.192)	0.122 (0.062-0.171)	0.116 (0.063-0.175)	0.128 (0.063-0.195)	0.077 (0.047-0.110)	0.074 (0.049-0.100)	0.068 (0.027-0.116)	0.078 (0.056-0.099)	0.064 (0.046-0.078)	0.059 (0.045-0.070)	0.053 (0.037-0.066)
Recovery probability outside Germany	0.074 (0.014-0.137)	0.095 (0.016-0.177)	0.108 (0.005-0.272)	0.096 (0.021-0.168)	0.051 (0.005-0.103)	0.067 (0.005-0.157)	0.041 (0.006-0.075)	0.100 (0.012-0.190)	0.020 (0.001-0.054)	0.020 (0.001-0.055)	0.014 (0.001-0.046)	0.011 (0.000-0.029)

TABLE S2. Posterior probability per spatial unit that the temporal range in survival (2000-2022) for one age class exceeds that of another (obtained from model M3). See Table 1 for the unit codes. Juv: juvenile survival, 1y: survival of 1y individuals, 2y: survival of 2y individuals, Ad: survival of >=3y individuals.

Region	Juv > 1y	Juv > 2y	Juv > Ad	1y > 2y	1y > Ad	2y > Ad
**BW**	0.772	0.716	0.985	0.409	0.717	0.846
**RP**	0.987	0.979	1.000	0.409	0.821	0.880
**BY**	0.993	0.962	0.996	0.393	0.421	0.560
**HE**	0.959	0.798	0.997	0.254	0.602	0.842
**NW**	0.137	0.067	0.217	0.340	0.673	0.833
**TH**	0.356	0.175	0.389	0.275	0.519	0.724
**NI**	0.191	0.590	0.323	0.851	0.711	0.250
**SH**	0.518	0.559	0.984	0.531	0.927	0.922
**ST**	0.304	0.030	0.526	0.107	0.728	0.983
**SN**	0.811	0.686	0.975	0.372	0.782	0.868
**MV**	0.408	0.058	0.831	0.124	0.832	0.982
**BB**	0.550	0.596	0.931	0.540	0.758	0.737
